# Supplementary material for: Prevalence, pattern and determinants of chronic disease multimorbidity in Nepal: secondary analysis of a national survey
Source: BMJ Open. 2021 Jul 27;11(7):e047665. doi: 10.1136/bmjopen-2020-047665 (PMC8317126; doi:10.1136/bmjopen-2020-047665)
Supplement: Supplementary data [file bmjopen-2020-047665supp001.pdf]

**Operational definitions of the key study variables**

| Variables | Definitions |
|---|---|
| Smoking | Participants were asked with two questions 'Have you smoked at least 100 cigarettes in your entire life?" and "Do you now smoke cigarettes every day, some days, or not at all?" Smoking at least 100 cigarettes in their lifetime and who, at the time of the survey, smoke either every day or some days was considered smoking (current). |
| Alcohol | Consuming alcohol in the past 30 days was considered alcohol consumption (current) |
| Bodyweight | **Body mass index (BMI)** was categorized as underweight (BMI<18.5) normal (BMI 18.0–24.9 kg/m$^2$), overweight (BMI 25.0–29.9 kg/m$^2$), or obesity (BMI ≥30 kg/m$^2$) |
| Non-HDL | An optimal level of non-HDL cholesterol is considered as having less than 130 milligrams per deciliter (mg/dL) |
| Hypertension | **Hypertension was** defined as having systolic blood pressure ≥140 mm Hg and/or diastolic blood pressure ≥90 mm Hg during the study, or being previously diagnosed as having hypertension determined by sighting documentation such as a treatment record book or by the history of the participant taking medicine for high blood pressure |
| Diabetes | Raised fasting glucose (≥126mg/dl) and/or raised postprandial blood glucose level ((≥200mg/dl) and/or if there is the current use of medications for diabetes |
| Coronary artery diseases (CAD) | Coronary artery disease will be defined as<br>(a) Definite CAD based on any of: documented evidence of prior acute coronary syndrome (ACS) or treatment for CAD, documented history of undergoing coronary angioplasty or CABG, more than 50 % epicardial coronary stenosis by invasive coronary angiography, ECG showing pathological Q waves (any of Minnesota code 1-1-1 to 1-1-7 or 1-2-1 to 1-2-5 or 1-2-7), imaging evidence of a region of loss of viable myocardium that is thinned and has a motion abnormality, in the absence of a non-ischemic cause, RAQ angina plus ECG changes (any of Minnesota codes 4-1-1, 4-1-2, 4-2 or 5-l, 5-2), or RAQ angina plus positive treadmill ECG (exercise-induced horizontal or down-sloping ST depression of ≥ 1 mm at 80 ms from J point), or inducible ischemia on stress imaging;<br>(b) Probable CAD based on any of (in the absence of any of the definite criteria): RAQ angina without significant ECG changes, ECG changes (any of Minnesota Code 4-1-1,4-1-2, 4-2 or 5-1, 5-2) without RAQ angina, or positive treadmill ECG without RAQ angina.<br>(c) Any CAD as those who satisfied either definite or probable CAD criteria. |
| Chronic Obstructive Pulmonary Disease (COPD) | COPD is the presence of a post-bronchodilator $FEV_1$/ FVC < 0.70 |
| Chronic Kidney | CKD is defined as glomerular filtration rate <60ml/min/1.73m$^2$ for three months or more irrespective of the cause.<br>or |

| | |
|---|---|
| Disease (CKD) | by the presence of albuminuria; urinary albumin: creatinine ratio > 30mg/gm to 30 to 300 mg/ gm in two of the three (1 to 2 weeks apart) spot urine specimens |
| Cancer (CA) | Self-reported cases of any types of cancer |
