# Supplementary material for: Prevalence, pattern and determinants of chronic disease multimorbidity in Nepal: secondary analysis of a national survey
Source: BMJ Open. 2021 Jul 27;11(7):e047665. doi: 10.1136/bmjopen-2020-047665 (PMC8317126; doi:10.1136/bmjopen-2020-047665)
Supplement: Supplementary data [file bmjopen-2020-047665supp002.pdf]

Table 1. Clustering of NCD risk factors by socio-demographic characteristics (multivariable Poisson regression analysis)

Variables		Adjusted prevalence ratio	*p* -value	95% CI
Gender	Female			
	Male	1.13	0.00	1.05 - 1.23
Age groups	<30 years			
	30-44 years	1.92	0.00	1.63 - 2.26
	45-59 years	3.21	0.00	2.73 - 3.79
	60-74 years	4.26	0.00	3.60 - 5.04
	≥75 years	5.50	0.00	4.56 - 6.64
Marital status	Never married			
	Currently married/Cohabiting	0.91	0.45	0.73 - 1.15
	Separated/Divorced	0.75	0.18	0.48 - 1.15
	Widowed	0.92	0.53	0.72 - 1.19
Ethnicity	Dalit			
	Disadvantaged Janajatis	1.29	0.00	1.17 - 1.43
	Disadvantaged non-Dalit Terai caste groups	1.08	0.10	0.99 - 1.18
	Religious minorities	1.28	0.00	1.12 - 1.47
	Relatively advantaged Janajatis	1.20	0.15	0.94 - 1.55
	Upper caste groups	1.15	0.00	1.06 - 1.26
Religion	Hindu			
	Buddhist	0.93	0.30	0.81 - 1.07
	Muslim	1.12	0.27	0.91 - 1.38
	Christian	1.03	0.84	0.77 - 1.39
	Kirat	1.03	0.81	0.82 - 1.29
Education	No education			
	primary education	1.12	0.11	0.97 - 1.29
	lower secondary education	1.16	0.05	1.00 - 1.35
	secondary education	1.15	0.10	0.97 - 1.35
	intermidiate or plus 2	1.08	0.29	0.94 - 1.25
	graduate and above	1.11	0.17	0.96 - 1.30
Occupation	Government employee			
	Non-government employee	0.89	0.29	0.72 - 1.11
	Self-employed/ Business	0.92	0.36	0.77 - 1.10
	Agriculture (commercial)	0.82	0.03	0.69 - 0.98
	Labour	0.78	0.02	0.63 - 0.96
	Student	0.74	0.19	0.46 - 1.17
	Homemaker	0.94	0.49	0.79 - 1.12
	Unemployed	1.00	0.98	0.82 - 1.21
	Retired	1.00	0.98	0.83 - 1.20
Income quintile	Lowest			
	Second	1.01	0.87	0.92 - 1.10
	Middle	1.04	0.40	0.94 - 1.16
	Fourth	1.11	0.03	1.01 - 1.22
	Highest	1.04	0.41	0.95 - 1.14

Province	Province 1			
	Province 2	1.21	0.01	1.04 - 1.40
	Bagmati	1.18	0.01	1.04 - 1.34
	Gandaki	1.04	0.53	0.91 - 1.19
	Lumbini	1.17	0.02	1.02 - 1.33
	Karnali	1.23	0.05	1.00 - 1.50
	Sudurpaschim	1.13	0.16	0.95 - 1.34
Residence	Rural			
	Urban	1.11	0.00	1.04 - 1.20
Smoking	No			
	Yes	0.97	0.41	0.90 - 1.04
ohol consumption	No			
	Yes	1.13	0.00	1.05 - 1.20
Weight	Underweight (<18.5 kg/m2)			
	Normal (18.5-24.9 kg/m2)	0.93	0.17	0.84 - 1.03
	Overweight (25-29.9 kg/m2)	1.27	0.00	1.18 - 1.36
	Obese (≥30 kg/m2)	1.44	0.00	1.28 - 1.61
High non-HDL	No (<130 mg/dL)			
	Yes (≥130 mg/dL)	1.16	0.00	1.10 - 1.24
