# Supplementary material for: Prevalence, pattern and determinants of chronic disease multimorbidity in Nepal: secondary analysis of a national survey
Source: BMJ Open. 2021 Jul 27;11(7):e047665. doi: 10.1136/bmjopen-2020-047665 (PMC8317126; doi:10.1136/bmjopen-2020-047665)
Supplement: Supplementary data [file bmjopen-2020-047665supp003.pdf]

**Table 1. Hierarchical logistic regression models**						
	**Model 1**		**Model 2**		**Model 3**	
	**Odds ratio**	**95% CI**	**Odds ratio**	**95% CI**	**Odds ratio**	**95% CI**
**Level 1- Distant factors**						
Age groups						
<30 years	Reference					
30-44 years	10.17***	5.49-18.86	9.99***	5.38-18.55	9.45***	4.95-18.06
45-59 years	32.23***	17.09-60.76	31.94***	16.94-60.21	27.10***	13.88-52.89
60-74 years	58.54***	30.69-111.66	58.64***	30.70-112.00	52.78***	26.65-104.53
≥75 years	117.52***	60.33-228.91	117.39***	60.31-228.47	106.19***	52.93-213.02
Ethnicity						
Dalit	Reference					
Disadvantaged Janajatis	0.60**	0.44-0.83	0.58***	0.43-0.80	0.59**	0.43-0.82
Disadvantaged non-Dalit Terai caste	0.88	0.61-1.28	0.88	0.60-1.27	0.93	0.63-1.37
Religious minorities	1.15	0.69-1.91	1.18	0.71-1.96	1.19	0.69-2.04
Relatively advantaged Janajatis	0.79	0.57-1.07	0.76	0.56-1.04	0.72*	0.52-0.99
Upper caste groups	0.54***	0.40-0.73	0.55***	0.41-0.75	0.55***	0.40-0.74
Education						
No education	Reference					
Primary education	1.26	0.99-1.61	1.23	0.96-1.57	1.30*	1.01-1.68
Lower secondary education	1.08	0.79-1.47	1.05	0.77-1.44	1.02	0.74-1.41
Secondary education	1.31*	1.01-1.70	1.27	0.98-1.64	1.22	0.94-1.59
Intermediate or plus 2	1.82**	1.25-2.65	1.75**	1.20-2.54	1.65*	1.12-2.44
Graduate and above	1.08	0.72-1.62	1.04	0.69-1.57	1.01	0.66-1.54
Wealth quintile						
Lowest	Reference					
Second	1.08	0.83-1.40	1.06	0.82-1.38	1.04	0.80-1.36
Middle	1.25	0.96-1.61	1.24	0.96-1.61	1.17	0.89-1.53
Fourth	1.42**	1.13-1.80	1.40**	1.10-1.77	1.33*	1.04-1.71
Highest	1.33*	1.04-1.71	1.31*	1.02-1.68	1.22	0.94-1.59
Province						
Province 1	Reference					
Province 2	1.38	0.94-2.02	1.42	0.97-2.08	1.44	0.97-2.13
Bagmati	1.48*	1.06-2.07	1.51*	1.08-2.11	1.4	1.00-1.97
Gandaki	0.88	0.61-1.29	0.89	0.61-1.30	0.81	0.55-1.20
Lumbini	1.18	0.83-1.66	1.18	0.84-1.67	1.14	0.81-1.62
Karnali	1.22	0.73-2.04	1.27	0.76-2.12	1.37	0.83-2.27
Sudurpaschim	1.13	0.75-1.69	1.17	0.79-1.75	1.2	0.80-1.80
Residence						
Rural	Reference					
Urban	1.39***	1.16-1.66	1.39***	1.16-1.66	1.29**	1.07-1.56
**Level 2- Intermediate factors**						
Smoking						
No	Reference					

Yes			0.80*	0.66-0.96	0.86	0.71-1.05
Alcohol Consumption						
No	Reference					
Yes			1.26*	1.04-1.51	1.28*	1.06-1.55
**Level 3- Immediate factors**						
Bodyweight						
Underweight	Reference					
Normal					1.06	0.79-1.42
Overweight					1.72***	1.26-2.34
Obese					2.35***	1.54-3.59
High non-HDL						
No	Reference					
Yes					1.23*	1.03-1.45

Note: *, p <0.05; **, p<0.01; ***, p<0.001
