# Supplementary material for: Prevalence, pattern and determinants of chronic disease multimorbidity in Nepal: secondary analysis of a national survey
Source: BMJ Open. 2021 Jul 27;11(7):e047665. doi: 10.1136/bmjopen-2020-047665 (PMC8317126; doi:10.1136/bmjopen-2020-047665)
Supplement: Author's manuscript [file bmjopen-2020-047665.draft_revisions.pdf]

BMJ Open

BMJ Open is committed to open peer review. As part of this commitment we make the peer review history of every article we publish publicly available.

When an article is published we post the peer reviewers' comments and the authors' responses online. We also post the versions of the paper that were used during peer review. These are the versions that the peer review comments apply to.

The versions of the paper that follow are the versions that were submitted during the peer review process. They are not the versions of record or the final published versions. They should not be cited or distributed as the published version of this manuscript.

BMJ Open is an open access journal and the full, final, typeset and author-corrected version of record of the manuscript is available on our site with no access controls, subscription charges or pay-per-view fees (http://bmjopen.bmj.com).

If you have any questions on BMJ Open's open peer review process please email info.bmjopen@bmj.com

Prevalence, pattern, distribution and determinants of chronic disease multimorbidity among adults in Nepal: secondary analysis of a national survey

Journal:	*BMJ Open*
Manuscript ID	bmjopen-2020-047665
Article Type:	Original research
Date Submitted by the Author:	07-Dec-2020
Complete List of Authors:	Dhungana, Raja Ram; Nepal Family Development Foundation<br>Karki, Khem; Ministry of Health and Population<br>Bista, Bihungum; Nepal Health Research Council<br>Pandey, Achyut; Nepal Health Sector Programme 3 / Monitoring, Evaluation and Operational Research, Abt Associates, Kathmandu, Nepal<br>Dhimal, Meghnath; Nepal Health Research Council<br>Maskey, Mahesh; Nepal Public Health Foundation
Keywords:	Hypertension < CARDIOLOGY, General diabetes < DIABETES & ENDOCRINOLOGY, Chronic renal failure < NEPHROLOGY

SCHOLARONE™
Manuscripts

I, the Submitting Author has the right to grant and does grant on behalf of all authors of the Work (as defined in the below author licence), an exclusive licence and/or a non-exclusive licence for contributions from authors who are: i) UK Crown employees; ii) where BMJ has agreed a CC-BY licence shall apply, and/or iii) in accordance with the terms applicable for US Federal Government officers or employees acting as part of their official duties; on a worldwide, perpetual, irrevocable, royalty-free basis to BMJ Publishing Group Ltd ("BMJ") its licensees and where the relevant Journal is co-owned by BMJ to the co-owners of the Journal, to publish the Work in this journal and any other BMJ products and to exploit all rights, as set out in our licence.

The Submitting Author accepts and understands that any supply made under these terms is made by BMJ to the Submitting Author unless you are acting as an employee on behalf of your employer or a postgraduate student of an affiliated institution which is paying any applicable article publishing charge ("APC") for Open Access articles. Where the Submitting Author wishes to make the Work available on an Open Access basis (and intends to pay the relevant APC), the terms of reuse of such Open Access shall be governed by a Creative Commons licence – details of these licences and which Creative Commons licence will apply to this Work are set out in our licence referred to above.

Other than as permitted in any relevant BMJ Author's Self Archiving Policies, I confirm this Work has not been accepted for publication elsewhere, is not being considered for publication elsewhere and does not duplicate material already published. I confirm all authors consent to publication of this Work and authorise the granting of this licence.

For peer review only

Prevalence, pattern, distribution and determinants of chronic disease multimorbidity among adults in Nepal: secondary analysis of a national survey

Raja Ram Dhungana[1*], Khem Karki[2†], Bihungum Bista[3], Achyut Raj Pandey[4], Meghnath Dhimal[3], Mahesh Kumar Maskey[5]

[1]Nepal Family Development Foundation, Kathmandu, Nepal

[2]Ministry of Health and Population, Kathmandu, Nepal

[3]Nepal Health Research Council, Kathmandu Nepal

[4]Nepal Health Sector Programme 3 / Monitoring, Evaluation and Operational Research, Abt Associates, Kathmandu, Nepal

[5]Nepal Public Health Foundation, Kathmandu, Nepal

[*], Correspondence: Dr Raja Ram Dhungana. Nepal Family Development Foundation, Nepal

Email: raja.dhungana@gmail.com

[†], Co-first author

For peer review only

For peer review only - http://bmjopen.bmj.com/site/about/guidelines.xhtml

ABSTRACT:

Objectives: To assess the prevalence, pattern, distribution and determinants of noncommunicable diseases (NCDs) multimorbidity in Nepal.

Design: Secondary analysis of the data from the NCD survey 2018, which was conducted between 2016-2018.

Setting: The data belong to the nationally representative survey, that selected the study samples from throughout Nepal using multi-stage cluster sampling.

Participants: 8931 participants aged 20 years and older were included in the study.

Primary Outcomes: NCD multimorbidity (occurrence of two or more chronic conditions including hypertension, diabetes, chronic obstructive pulmonary disease, chronic kidney disease, coronary artery disease, and cancer). Descriptive statistics, concentration indices, and odds ratios were computed to assess pattern, distribution and determinants of multimorbidity.

Results: Mean (standard deviation) age was 46.7 years (14.9 years). The majority of the participants were female (57.8%), without formal education (53.4%) and from urban areas (51.5%). Multimorbidity was present in 13.96% (95% CI: 12.9-15.1). Hypertension and diabetes coexisted in 5.7%. Multimorbidity (concentration index (ConI) = 0.05), hypertension (ConI=0.09) and diabetes (ConI=0.04) had pro-rich income inequality. Age, alcohol consumption, body mass index, non-HDL level and rural-urban setting were significantly associated with multimorbidity.

Conclusion: Multimorbidity was prevalent in particular groups or geographical areas in Nepal suggesting a need for coordinated and integrated NCD care approach for the management of multiplicative co-comorbid conditions.

Keywords: Multimorbidity, comorbidity, hypertension, diabetes, chronic obstructive pulmonary disease, chronic kidney disease, coronary artery disease, cancer, Nepal

STRENGTH

- This study is the first to assess the prevalence, pattern, distribution and determinants of NCD multimorbidity in Nepal

For peer review only - http://bmjopen.bmj.com/site/about/guidelines.xhtml

- Data were collected from the national representative sample of 20 years and older adults in Nepal

LIMITATIONS

- Data relating to cancer are prone to self-reporting bias
- Estimating the multimorbidity from only six NCDs is likely to underestimate its condition in Nepal

For peer review only

For peer review only - http://bmjopen.bmj.com/site/about/guidelines.xhtml

BACKGROUND

Globally, noncommunicable diseases (NCDs) are giving rise to enormous public health challenges. Recently, the co-existence of two and more NCDs (multimorbidity) has escalated the burden of NCDs globally [1-6]. The prevalence of multimorbidity varies from 12% to 95% across different countries [7-9], clustering particularly in socioeconomically deprived areas [10].

Effects of multimorbidity range from an individual to the health system level. In an individual, effects can stretch out from poor physical and mental health outcomes to high treatment burden for the management of multiple chronic diseases [2 11]. Likewise, multiplications of the comorbid conditions could exert enamours pressure to the existing model of healthcare delivery that lacks coordination and integration of NCD services by increasing healthcare costs and workload [8 11 12].

The major NCDs shared the common behavioural and metabolic risk factors such as physical inactivity, smoking, alcohol consumption and low fruit and vegetables and obesity, which are the leading contributors to burden of multimorbidity [13 14]. Evidence shows that various social circumstances associated with income, education, occupation, gender, and ethnicity also determine the distribution of NCDs and multimorbidity [2 9 15 16]. For example, multimorbidity comes about much earlier in life, up to 15 years earlier, among the people belonging to the poor socio-economic status compared to their wealthier counterparts [4 17]. Additionally, ageing, advancement of healthcare, and increasing life expectancy are closely linked with NCDs and thus are also related to multimorbidity [2 15].

Recent epidemiological surveys indicated an increasing trend of prevalence of chronic conditions including hypertension and diabetes in Nepal [18]. For example, hypertension increased from 21% to 25% between 2007 [19] and 2019 [18], and prevalence of diabetes rose from 3.6% to 5.8% between 2013 [20] and 2019 [18]. While the prevalence of NCDs is in rising trend, more individuals are likely to acquire multimorbidity. However, no systematic investigation on NCD multimorbidity is available in Nepal. Detail epidemiological information is essential for facilitating health system preparedness and response in terms of prevention and management of multimorbidity. Therefore, this study aimed to assess prevalence, patterns, distribution and determinants of multimorbidity in Nepal.

METHODS

Data source, study participants and sampling

For peer review only - http://bmjopen.bmj.com/site/about/guidelines.xhtml

We used the data from the first nationally representative population-based noncommunicable diseases survey in Nepal (shortly, NCD survey) [21]. The cross-sectional survey was conducted between 2016 and 2018. The purpose of NCD survey was to estimate the prevalence of selected NCDs including hypertension (HTN), diabetes (DM), chronic obstructive pulmonary disease (COPD), Chronic kidney disease (CKD), coronary artery disease (CAD), and cancer (CA) in Nepal.

The NCD survey applied a multistage cluster sampling method to select the participants aged 20 years and above from throughout Nepal. Details of the sampling procedure and survey reports are available elsewhere [21 22]. For our study purpose, we included the data from 8931 participants those having information on six NCDs.

Data collection

The NCD survey collected information on HTN, DM, COPD, CKD, CAD and CA. The survey team recorded three readings of systolic and diastolic blood pressure and used the average of the last two measurements of blood pressure to define hypertension as the condition having systolic blood pressure ≥140 mmHg and/or diastolic blood pressure ≥190 mmHg and/or the use of antihypertensive medication. The survey considered the participants as diabetics if they had raised fasting glucose (≥126mg/dl) or raised postprandial blood glucose level ((≥200mg/dl) or the participant was on anti-diabetic medication. COPD was diagnosed based on the ratio of FEV1 to FVC and CKD was confirmed using urinary albumin to creatinine ratio (uACR) and/or measurement of serum creatinine/estimated glomerular filtration rate (e-GFR) [21]. For diagnosing CAD, the survey used the a) information on self-reported admission for myocardial infarction, percutaneous coronary angioplasty or coronary artery bypass surgery; b) electrocardiogram test; and c) responses on Rose Angina questionnaire [23]. Details are given in the survey report [21]. Cancer diagnosis was confirmed as informed by the participants. Details on operational definitions of the study variables are given in the survey report [21] and Supplementary file 1.

The NCD survey also collected information related to socio-demographic characteristics, household income, smoking, alcohol consumption, body mass index (BMI) and lipid. We extracted the data both on the outcome (HTN, DM, COPD, CKD, CAD and CA) and study variables (socio-demographic characteristics, smoking, alcohol consumption, overweight/obesity and high non-HDL) and defined them as per the survey criteria to assess the burden of NCD multimorbidity in Nepal. Multimorbidity was defined as the presence of two and more NCD disorders [10].

For peer review only - http://bmjopen.bmj.com/site/about/guidelines.xhtml

Data analysis

We used the NCD survey information on primary sample units, strata and sampling weight to construct the complex survey weight and performed data analysis using STATA software version 16.1 (Stata Corporation, College Station, TX, USA). All estimates are presented with 95% confidence intervals (CIs). Geographic distribution and clustering of NCDs are presented graphically. To better understand the independent effects of covariates on multimorbidity, we considered the number of NCDs occurred in each participant as count data and applied multiple Poisson regression and reported the adjusted relative risk (aRR) of having x number NCDs.

To assess the degree of income inequalities in NCD multimorbidity, we plotted a concentration curve using the cumulative percentage of each indicator (*y*-axis) against the cumulative percentage of household income quintiles (*x*-axis) [26]. Given the dichotomous nature of the outcome variables, we used Erreygers correction and specified the limits as 0 and 1 [24].

For multivariable analysis, we created the hierarchy of the variables as level 1 - distant factors (all socio-demographic variables: age, gender, marital status, ethnicity, religion, education, occupation, household income, Province, and residence), level 2 -intermediate factors (behavioural factors: smoking and alcohol consumption) and level 3- immediate factors (metabolic factors: body mass index and non-HDL) and applied hierarchical modelling using a series of stepwise (backward selection, selection criteria: p-value <0.2) logistic regression. For sensitivity analysis, we imputed the 568 missing data on non-HDL using logistic model and replicated the analysis like in the main analysis. A p-value <0.05 is considered as statistically significant.

Ethics

The NCD survey obtained ethical approval from the Ethical Review Board of Nepal Health Research Council. Informed consent was obtained from the study participants obtained ensuring voluntary participation, privacy and confidentiality.

Patient and public involvement

This is the secondary data analysis of the data from the NCD survey. Patients were not involved in the design or conduct of this study, and nor were members of the general public.

RESULTS

For peer review only - http://bmjopen.bmj.com/site/about/guidelines.xhtml

Sociodemographic characteristics

The mean (standard deviation) age was 46.7 years (14.9 years). The majority of the participants were female (57.8%), married (88.5%), Hindu (88.9%), without formal education (53.4%), and from urban areas (51.5%) (Table 1).

Table 1. Characteristics of the participants (N=8931)

**Categories**		**Male (%)**	**Female (%)**	**Total (%)**
Age groups	<30 years	5.83	9.51	15.34
	30-44 years	10.38	17.87	28.25
	45-59 years	15.79	16.84	32.63
	60-74 years	11.48	9.12	20.60
	≥75 years	2.12	1.05	3.18
Marital status	Never married	2.75	1.85	4.60
	Currently married/cohabiting	41.55	46.93	88.48
	Separated/Divorced	0.15	0.45	0.60
	Widowed	1.15	5.17	6.32
Ethnicity	Dalit	3.94	5.52	9.46
	Disadvantaged Janajatis	9.45	11.29	20.74
	Disadvantaged non-Dalit Terai caste	8.63	8.55	17.18
	Religious minorities	1.53	1.26	2.79
	Relatively advantaged Janajatis	7.90	9.35	17.25
	Upper caste groups	14.16	18.43	32.59
Religion	Hindu	40.27	48.64	88.91
	Buddhist	2.57	2.84	5.41
	Muslim	1.91	1.76	3.67
	Christian	0.32	0.44	0.76
	Kirat and other	0.54	0.71	1.25
Education	No education	17.10	36.25	53.35
	Primary education	7.34	3.93	11.27
	Lower secondary education	5.22	3.42	8.64
	Secondary education	8.31	5.67	13.98
	Intermediate or plus 2	3.59	2.95	6.54
	Graduate and above	4.05	2.18	6.22
Occupation	Government employee	2.72	1.10	3.82
	Non-government employee	2.95	1.31	4.27
	Self-employed/ Business	8.44	3.55	11.99
	Agriculture (commercial)	20.99	16.83	37.82
	Labour	3.13	0.85	3.99
	Student	1.24	0.98	2.23
	Homemaker	1.27	25.96	27.24
	Unemployed	2.10	3.19	5.29
	Retired	2.74	0.62	3.36
Income quintile	Lowest	9.78	12.47	22.25
	Second	8.59	10.38	18.96
	Middle	6.87	8.61	15.48

For peer review only

For peer review only - http://bmjopen.bmj.com/site/about/guidelines.xhtml

	Fourth	10.99	12.29	23.29
	Highest	9.60	10.42	20.02
Province	Province 1	7.49	9.30	16.78
	Province 2	9.37	9.03	18.40
	Bagmati	10.60	12.98	23.58
	Gandaki	3.92	5.51	9.43
	Lumbini	7.91	8.97	16.88
	Karnali	2.12	3.08	5.20
	Sudurpaschim	4.21	5.53	9.74
Residence	Rural	20.97	27.55	48.52
	Urban	21.19	30.29	51.48

Prevalence of NCD multimorbidity

The prevalence of NCD multimorbidity was 13.96% (95% CI: 12.9-15.1). Participants at older ages, males, religious minorities, those without formal education and obese had a higher prevalence of multimorbidity (Table 2).

Table 2. Prevalence of multimorbidity by subgroups (N=8931)

		Multimorbidity	
**Categories**		**Percent**	**95% CI**
Age groups	<30 years	0.81	0.44-1.5
	30-44 years	6.23	5.2-7.45
	45-59 years	16.75	15.1-18.54
	60-74 years	26.01	23.39-28.82
	≥75 years	39.56	32.94-46.6
Gender	Female	12.02	10.77-13.38
	Male	16.29	14.83-17.86
Marital status	Never married	2.89	1.48-5.56
	Currently married/cohabiting	13.9	12.79-15.09
	Separated/Divorced	14.03	6.74-26.93
	Widowed	22.71	18.98-26.92
Ethnicity	Dalit	14.26	11.77-17.17
	Disadvantaged Janajatis	11.4	9.79-13.24
	Disadvantaged non-Dalit Terai caste	14.69	12.36-17.38
	Religious minorities	21.14	15.98-27.41
	Relatively advantaged Janajatis	18.45	15.52-21.79
	Upper caste groups	12.14	10.64-13.83
Religion	Hindu	13.66	12.54-14.87
	Buddhist	15.29	11.53-20
	Muslim	20.06	15.49-25.58
	Christian	9.34	4.17-19.63
	Kirat and other	14.49	8.25-24.21
Education	No education	15.85	14.45-17.36
	Primary education	14.32	11.9-17.13

For peer review only - http://bmjopen.bmj.com/site/about/guidelines.xhtml
For peer review only

	Lower secondary education	10.8	8.38-13.81
	Secondary education	11.91	9.72-14.52
	Intermediate or plus 2	10.99	8.15-14.66
	Graduate and above	9.28	6.87-12.42
Occupation	Government employee	16.69	12.29-22.26
	Non-government employee	12.79	9.07-17.75
	Self-employed/ Business	15.98	12.95-19.56
	Agriculture(commercial)	11.78	10.37-13.34
	Labour	10.23	7.36-14.03
	Student	0.37	0.09-1.47
	Homemaker	14.81	13.01-16.83
	Unemployed	18.27	14.35-22.98
	Retired	29.71	24.55-35.45
Income quintile	Lowest	11.5	9.91-13.32
	Second	11.73	9.97-13.75
	Middle	13.2	11.16-15.55
	Fourth	16.96	14.83-19.32
	Highest	15.87	13.75-18.24
Residence	Rural	10.89	9.69-12.23
	Urban	17.03	15.32-18.89
Smoking (current)	No	13.9	12.71-15.19
	Yes	14.17	12.37-16.19
Alcohol consumption (current)	No	13.28	12.11-14.56
	Yes	15.91	14.08-17.92
Weight	Underweight (<18.5 kg/m2)	12.26	9.82-15.21
	Normal (18.5-24.9 kg/m2)	10.91	9.87-12.05
	Overweight (25-29.9 kg/m2)	20.74	18.32-23.38
	Obese (≥30 kg/m2)	30.28	25.48-35.55
High non-HDL	No (<130 mg/dL)	11.5	10.4-12.7
	Yes (≥130 mg/dL)	18.73	16.87-20.75

In geographic distribution, Bagmati province had the highest proportion of multimorbidity (Fig 1). The prevalence of multimorbidity also varied by urban and rural settings, where more participants from urban than rural had multimorbidity.

Pattern of multimorbidity

Among the participants having multimorbidity, 80.1% had two NCDs, 17.4% were with three NCDs and 2.5% reported having four and more NCDs. The clustering of NCDs significantly varied with age and gender (Fig 2), where NCDs were more likely to occur at old ages and in males (Supplementary file 2).

For peer review only

For peer review only - http://bmjopen.bmj.com/site/about/guidelines.xhtml

The most common pair of co-occurrences NCDs were hypertension and diabetes, followed by hypertension and COPD, and hypertension and CKD (Fig 3). The triad 'hypertension, diabetes and CKD' was present in 1.4%.

Income inequality in multimorbidity

The concentration index (ConI) for multimorbidity was 0.046 (p<0.001) indicating multimorbidity was concentrated among the richest. Hypertension (ConI=0.09, p<0.001) and diabetes (ConI=0.04, p<0.001) also had pro-rich income inequality. COPD (ConI= - 0.02, p=0.045) concentrated among the poorest. CAD (ConI=0.005, p=0.3) and CKD (ConI=-0.0002, p=0.97) also had pro-poor inequality, but not statistically significant (Fig 4).

Factors associated with multimorbidity

Age was significantly associated with NCD multimorbidity. The odds of having multimorbidity were 9.4 times for 30-44 years, 27.1 times for 45-59 years and 52.8 for 60-74 years compared to below 30 years of age group (Supplementary file 3). The probability of occurring multimorbidity was significantly higher in overweight/obesity, alcohol users, urban areas and those who had high non-HDL compared to others (Fig 5). The magnitude and direction of the estiamtes from the impuated dataset were similar to the main analysis.

DISCUSSION

Our study demonstrated the high burden of NCD multimorbidity in Nepal particularly affecting the elderly, obese, alcohol users, religious minorities, higher-income group, and urban residents. This is the very first findings on NCD multimorbidity in Nepal, which may inform the stakeholders on its status, patterns and distribution across Nepal.

The prevalence of NCD multimorbidity in our study was 13.96%. The finding is higher than the rate reported in South Asian counterparts (9.4%) [25], consistent with the prevalence reported in Canada (12.9%) [13], Switzerland (14.5%) [26] and China (14%) [27] and lower than that of Australia (47.1%) [28], Denmark (39.7%), Brazil (29%) [29], England (27.2%) [3], and Vietnam (16.4%) [30]. A systematic review that computed a pooled estimate of multimorbidity from 18 high-income countries and 31 low-and middle-income countries had found the prevalence of multimorbidity to be 33.1% [31]. As the prevalence of chronic conditions tends to differ across different countries owing to differences in dietary practices, lifestyle, alcohol consumption, tobacco use and level of physical activity, among others, it is

For peer review only - http://bmjopen.bmj.com/site/about/guidelines.xhtml

not uncommon for multimorbidity to vary across countries. Some of these disparities could be because of the differences in the methodology used in individual study. For example, prevalence of multimorbidity also depends on the number of diseases considered in the study, population under study and selection of participants.

The prevalence of multimorbidity was found to be slightly higher among males in our study. The prevalence of multimorbidity was 16% in males and 12% in females. After adjusting age, the difference in prevalence between males and females was not significant. Like our finding, one of the systematic reviews showed no statistically significant difference on the pooled prevalence of multimorbidity by gender though the nine of the studies those were reviewed reported a higher rate of multimorbidity in females than in males [9]. Likewise, the prevalence multimorbidity was 14.8% in male and 14.3% in female in Switzerland [26], and 17.2% in male and 15.5% in females in Vietnam, indicating no disparity in multimorbidity in-between [30].

Multiple studies conducted in different setting have revealed age as a determinant of multi-morbidity with the condition being more common with increasing age group [5 26 32]. The prevalence of multimorbidity was 0.81% among participants of age below 30 years and 39.5% in participants of age 75 years or above. In a similar study in Australia, the prevalence of multimorbidity was found to be 1.7% in participants of age 20-29 years, 33.6% among participants of age 70-79 years and 37.7% in participants of age 80 years which also indicates that the multimorbidity increases with increasing age [26]. In another study in the USA, the prevalence of multimorbidity was found to be 67% among those above 65 years and 71% among those above 75 years of age [33]. As most of the NCDs are more common in the older age group, there could have been the higher probability of co-occurrence and thus leading to multimorbidity.

In our study, the odds of having multimorbidity was approximately 3 folds higher in obese compared to normal-weight participants. Similar findings were also reported in a study by Agborsangaya et al. [14] where odds of having multimorbidity was 2 folds higher in obese. Higher risk of multimorbidity among the obese population could be because it is one of the most common risk factors shared among the NCDs.

We found that urban area and Bagmati province (capital) had a higher prevalence of multimorbidity. This could be linked to the high burden of NCD risk factors in those areas. Recent surveys suggested that smoking, alcohol consumption, physical inactivity, high salt

For peer review only - http://bmjopen.bmj.com/site/about/guidelines.xhtml

intake, and obesity were more prevalent in Bagmati province and urban areas than others [18 34].

The most common pair of co-occurrences NCDs were hypertension and diabetes (5.7%). The prevalence of comorbid diabetes and hypertension was twice than that we reported (2%) in 2013 in Nepal, suggesting an increasing burden of diabetes and hypertension comorbidity over the years [35]. The prevalence of comorbid diabetes and hypertension was found to be 4.5% in India [36] and 3% in Bangladesh [37]. Similarly, a study by Harrison et al in Australia had found that the co-occurrence of hypertension and diabetes was 4.1% [28]. The higher co-occurrence of diabetes and hypertension or the comorbid condition could be because both conditions are more prevalent in society and also share the common risk factors.

The concentration index (ConI) for multimorbidity was 0.046 ($p<0.001$) indicating multimorbidity was concentrated among the richest. Hypertension and diabetes had pro-high-income inequality in our study. Some of the previous studies have also indicated the higher prevalence of hypertension [38 39] and diabetes [18] among the richest segment of the population. Higher prevalence of these disease conditions and the multimorbidity among the richest segment could be associated with increased engagement in sedentary lifestyle in this particular group.

This is the first study to assess the burden of multimorbidity in Nepal. Findings from the nationally representative study could have a high policy implication in terms of tackling the growing burden of multimorbidity in Nepal. Our study found that NCD multimorbidity prevalent in Nepal. Higher prevalence of multimorbidity means more frequent hospital consultations and more hospitalizations [3 5 6]. For example, Schneider et al. [6] reported that the patients with three or more chronic conditions utilize 25 times more hospital beds than peers without any chronic conditions. As higher proportion of healthcare encounters are likely to occur because of multimorbidity, appropriate training of the health facilities at peripheral level and development of an effective referral system could be useful in resource constraint setting like Nepal.

Likewise, with the growing burden of multimorbidity, health system also needs to prepare for dealing with an additional number of health facility visits in Nepal. The current health system organization largely focuses on preventing and managing maternal and child health conditions. It requires a degree of reorganization to deal with the increasing burden of NCDs and multimorbidity. There are several evidence-based models are in practice for the

For peer review only - http://bmjopen.bmj.com/site/about/guidelines.xhtml

For peer review only

management of multimorbidity globally. Sustainable intEgrated chronic care modeLs for multi-morbidity: delivery, FInancing, and performance (SELFIE) is an example of an innovative framework that suggests for the integration of care at micro, meso and macro level according to six pillars of health system: service delivery, leadership and governance, workforce, financing, technologies and medical products and information and research [40]. The strategies, however, should be specific to Nepalese context and backed by evidence on health benefit and cost-effectiveness as the most common comorbid conditions could vary from one country to another.

This study has some limitations. The use of self-reported data for cancer diagnosis might have underestimated the prevalence of cancer in the original study, affecting the current study findings. Likewise, our study could not include other several comorbid conditions such as mental disorders and degenerative diseases and possibly underestimated the burden of multimorbidity in Nepal. For that, a national representative survey encompassing the majority of the common NCDs is recommended.

For peer review only

CONCLUSIONS

This study demonstrated that NCD multimorbidity is prevalent and disproportionately distributed across different socio-economic and demographic strata in Nepal. Growing prevalence of multimorbidity indicates an urgency for the health system to prepare and respond to the inevitable health threats and economic burden in Nepal.

COMPETING INTEREST

None declared.

AUTHORS' CONTRIBUTIONS

RRD and KK conceptualized the study. RRD analysed the data. RRD, BB and ARP interpreted the findings and prepared the first draft of the manuscript. KK, MD and MKM interpreted the findings and drafted the manuscript. All authors read and approved the final manuscript.

FUNDING

For peer review only - http://bmjopen.bmj.com/site/about/guidelines.xhtml

295 The authors received no specific grant from any funding agency for this work.

296 **ACKNOWLEDGMENTS**

297 We would like to thank Nepal Health Research Council for sharing the data. We appreciate
298 all the participants for their great contribution to the study by sharing the information.

299 **DATA SHARING STATEMENT**

300 Data used in the study are reposited in Nepal Health Research Council and are available from
301 the council upon a reasonable request to the Council.

302

303 **REFERENCE**

304

305 1. Wild S, Roglic G, Green A, Sicree R, King H. Global prevalence of diabetes: estimates for
306 the year 2000 and projections for 2030. Diabetes care 2004;**27**(5):1047-53
307 2. Koroukian SM, Schiltz NK, Warner DF, et al. Multimorbidity: constellations of conditions
308 across subgroups of midlife and older individuals, and related Medicare expenditures.
309 Journal of comorbidity 2017;**7**(1):33-43
310 3. Cassell A, Edwards D, Harshfield A, et al. The epidemiology of multimorbidity in primary
311 care: a retrospective cohort study. British Journal of General Practice
312 2018;**68**(669):e245-e51
313 4. Huntley AL, Johnson R, Purdy S, Valderas JM, Salisbury C. Measures of multimorbidity
314 and morbidity burden for use in primary care and community settings: a systematic
315 review and guide. The Annals of Family Medicine 2012;**10**(2):134-41
316 5. Salisbury C, Johnson L, Purdy S, Valderas JM, Montgomery AA. Epidemiology and
317 impact of multimorbidity in primary care: a retrospective cohort study. British Journal
318 of General Practice 2011;**61**(582):e12-e21
319 6. Schneider KM, O'Donnell BE, Dean D. Prevalence of multiple chronic conditions in the
320 United States' Medicare population. Health and quality of life outcomes 2009;**7**(1):82
321 7. Garin N, Koyanagi A, Chatterji S, et al. Global multimorbidity patterns: a cross-sectional,
322 population-based, multi-country study. Journals of Gerontology Series A: Biomedical
323 Sciences and Medical Sciences 2016;**71**(2):205-14
324 8. Oni T, McGrath N, BeLue R, et al. Chronic diseases and multi-morbidity-a conceptual
325 modification to the WHO ICCC model for countries in health transition. BMC public
326 health 2014;**14**(1):1-7
327 9. Violan C, Foguet-Boreu Q, Flores-Mateo G, et al. Prevalence, determinants and patterns of
328 multimorbidity in primary care: a systematic review of observational studies. PloS
329 one 2014;**9**(7):e102149
330 10. Barnett K, Mercer SW, Norbury M, Watt G, Wyke S, Guthrie B. Epidemiology of
331 multimorbidity and implications for health care, research, and medical education: a
332 cross-sectional study. The Lancet 2012;**380**(9836):37-43

For peer review only - http://bmjopen.bmj.com/site/about/guidelines.xhtml

11. Thavorn K, Maxwell CJ, Gruneir A, et al. Effect of socio-demographic factors on the association between multimorbidity and healthcare costs: a population-based, retrospective cohort study. BMJ open 2017;**7**(10):e017264
12. Wolff JL, Starfield B, Anderson G. Prevalence, expenditures, and complications of multiple chronic conditions in the elderly. Archives of internal medicine 2002;**162**(20):2269-76
13. Roberts K, Rao D, Bennett T, Loukine L, Jayaraman G. Prevalence and patterns of chronic disease multimorbidity and associated determinants in Canada. Health promotion and chronic disease prevention in Canada: research, policy and practice 2015;**35**(6):87
14. Agborsangaya CB, Ngwakongnwi E, Lahtinen M, Cooke T, Johnson JA. Multimorbidity prevalence in the general population: the role of obesity in chronic disease clustering. BMC Public Health 2013;**13**(1):1161
15. Wagner K-H, Brath H. A global view on the development of non communicable diseases. Preventive Medicine 2012;**54**:S38-S41 doi: https://doi.org/10.1016/j.ypmed.2011.11.012[published Online First: Epub Date]|.
16. Alwan A. *Global status report on noncommunicable diseases 2010*: World Health Organization, 2011.
17. Mercer SW, Zhou Y, Humphris GM, et al. Multimorbidity and socioeconomic deprivation in primary care consultations. The Annals of Family Medicine 2018;**16**(2):127-31
18. Dhimal M, Bista B, Saroj B, et al. Non communicable diseases risk factors: STEPS Survey Nepal 2019: Nepal Health Research Council (NHRC), 2019.
19. World Health Organisation. STEPwise approach to chronic disease risk factor surveillance: 2007 STEPS Survey report. Secondary STEPwise approach to chronic disease risk factor surveillance: 2007 STEPS Survey report 2008. https://www.who.int/ncds/surveillance/steps/nepal/en/.
20. Aryal KK, Neupane S, Mehata S, et al. Non communicable diseases risk factors: STEPS Survey Nepal 2013. Kathmandu, Nepal: Nepal Health Research Council, 2014.
21. Nepal Health Research Council. Population based prevalence of selected non-Communicable diseases in Nepal. Kathmandu, Nepal, 2019.
22. Dhimal M, Karki KB, Sharma SK, et al. Prevalence of Selected Chronic Non-Communicable Diseases in Nepal. J Nepal Health Res Counc 2019;**17**(3):394-401 doi: 10.33314/jnhrc.v17i3.2327[published Online First: Epub Date]|.
23. Poudel M, Bhandari R, Malla G, et al. Rose Angina Questionnaire: Validation in emergency department to detect myocardial infarction in a tertiary hospital of eastern Nepal. Hong Kong Journal of Emergency Medicine 2020;**27**(5):257-61
24. Erreygers G. Correcting the Concentration Index. Journal of Health Economics 2009;**28**(2):504-15 doi: https://doi.org/10.1016/j.jhealeco.2008.02.003[published Online First: Epub Date]|.
25. Singh K, Patel SA, Biswas S, et al. Multimorbidity in South Asian adults: prevalence, risk factors and mortality. Journal of public health (Oxford, England) 2019;**41**(1):80-89 doi: 10.1093/pubmed/fdy017[published Online First: Epub Date]|.
26. Rizza A, Kaplan V, Senn O, Rosemann T, Bhend H, Tandjung R. Age-and gender-related prevalence of multimorbidity in primary care: the Swiss FIRE project. BMC family practice 2012;**13**(1):113
27. Ruel G, Levesque J-F, Stocks N, et al. Understanding the evolution of multimorbidity: evidences from the North West Adelaide Health Longitudinal Study (NWAHS). PLoS One 2014;**9**(5):e96291

For peer review only - http://bmjopen.bmj.com/site/about/guidelines.xhtml

28. Harrison C, Henderson J, Miller G, Britt H. The prevalence of complex multimorbidity in Australia. Australian and New Zealand journal of public health 2016;**40**(3):239-44
29. Araujo MEA, Silva MT, Galvao TF, Nunes BP, Pereira MG. Prevalence and patterns of multimorbidity in Amazon Region of Brazil and associated determinants: a cross-sectional study. BMJ open 2018;**8**(11)
30. Ba NV, Minh HV, Quang LB, et al. Prevalence and correlates of multimorbidity among adults in border areas of the Central Highland Region of Vietnam, 2017. Journal of comorbidity 2019;**9**:2235042X19853382
31. Nguyen H, Manolova G, Daskalopoulou C, Vitoratou S, Prince M, Prina AM. Prevalence of multimorbidity in community settings: A systematic review and meta-analysis of observational studies. J Comorb 2019;**9**:2235042X19870934 doi: 10.1177/2235042X19870934[published Online First: Epub Date]|.
32. Pefoyo AJK, Bronskill SE, Gruneir A, et al. The increasing burden and complexity of multimorbidity. BMC public health 2015;**15**(1):415
33. Jindai K, Nielson CM, Vorderstrasse BA, Quiñones AR. Peer Reviewed: Multimorbidity and Functional Limitations Among Adults 65 or Older, NHANES 2005–2012. Preventing chronic disease 2016;**13**
34. Ministry of Health, New ERA, ICF. Nepal Demographic and Health Survey 2016. Kathmandu, Nepal: Ministry of Health, Nepal, 2017.
35. Pandey AR, Karki KB, Mehata S, et al. Prevalence and determinants of comorbid diabetes and hypertension in Nepal: evidence from non communicable disease risk factors STEPS Survey Nepal 2013. Journal of Nepal Health Research Council 2015;**13**(29):20-25
36. Tripathy JP, Thakur JS, Jeet G, Jain S. Prevalence and determinants of comorbid diabetes and hypertension: Evidence from non communicable disease risk factor STEPS survey, India. Diabetes & Metabolic Syndrome: Clinical Research & Reviews 2017;**11**:S459-S65
37. Biswas T, Townsend N, Islam MS, et al. Association between socioeconomic status and prevalence of non-communicable diseases risk factors and comorbidities in Bangladesh: findings from a nationwide cross-sectional survey. BMJ open 2019;**9**(3):e025538
38. Mishra SR, Ghimire S, Shrestha N, Shrestha A, Virani SS. Socio-economic inequalities in hypertension burden and cascade of services: nationwide cross-sectional study in Nepal. Journal of human hypertension 2019;**33**(8):613-25
39. Gupta R, Kaur M, Islam S, et al. Association of household wealth index, educational status, and social capital with hypertension awareness, treatment, and control in South Asia. American journal of hypertension 2017;**30**(4):373-81
40. Leijten FRM, Struckmann V, van Ginneken E, et al. The SELFIE framework for integrated care for multi-morbidity: development and description. Health policy 2018;**122**(1):12-22

FIGURES

Fig 1. Distribution of multimorbidity by province

Fig 2. Clustering of NCDs by gender

Fig 3. Co-occurrence of NCDs

For peer review only - http://bmjopen.bmj.com/site/about/guidelines.xhtml

Note: Co-occurrence of six major NCDs given in percent; HTN, hypertension; DM, diabetes; COPD, chronic obstructive pulmonary disease; CKD, Chronic kidney disease; CAD, coronary artery disease

Fig 4. Concentration curves

Note: y axis is the cumulative proportion of the outcome variables, x axis is the cumulative percentage of population ordered by income quintile beginning from lowest to higher quintile; HTN, hypertension; DM, diabetes; COPD, chronic obstructive pulmonary disease; CKD, Chronic kidney disease; CAD, coronary artery disease; CA, cancer.   The concentration index (ConI) is defined as twice the area between the concentration curve and the line of equality (the 45-degree blue line). A positive ConI (curve below the line of equality) indicates that (ill/ undesired) health is more prevalent among the rich, and a negative one (curve above the line of equality) tells that the outcome is more prevalent in the poor.

Fig 5. Factor associated with multimorbidity

Note: Odds ratio were estimated from the hierarchical logistic regression models. Details of output are given on Sumplementary file 2.

SUPPLEMENTARY MATERIALS

Supplementary file 1:

Operational definitions. (DOCX 16.9 KB)

Supplementary file 2:

Clustering of NCDs. (xlsx 13 KB)

Supplementary file 3:

Hierarchical logistic regression models. (xlsx 13 KB)

For peer review only

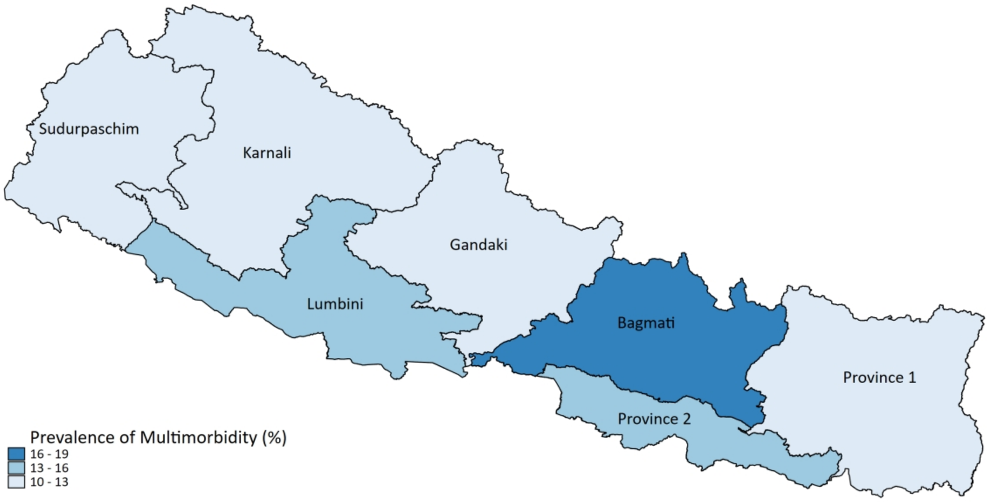

Fig 1. Distribution of multimorbidity by province

242x122mm (300 x 300 DPI)

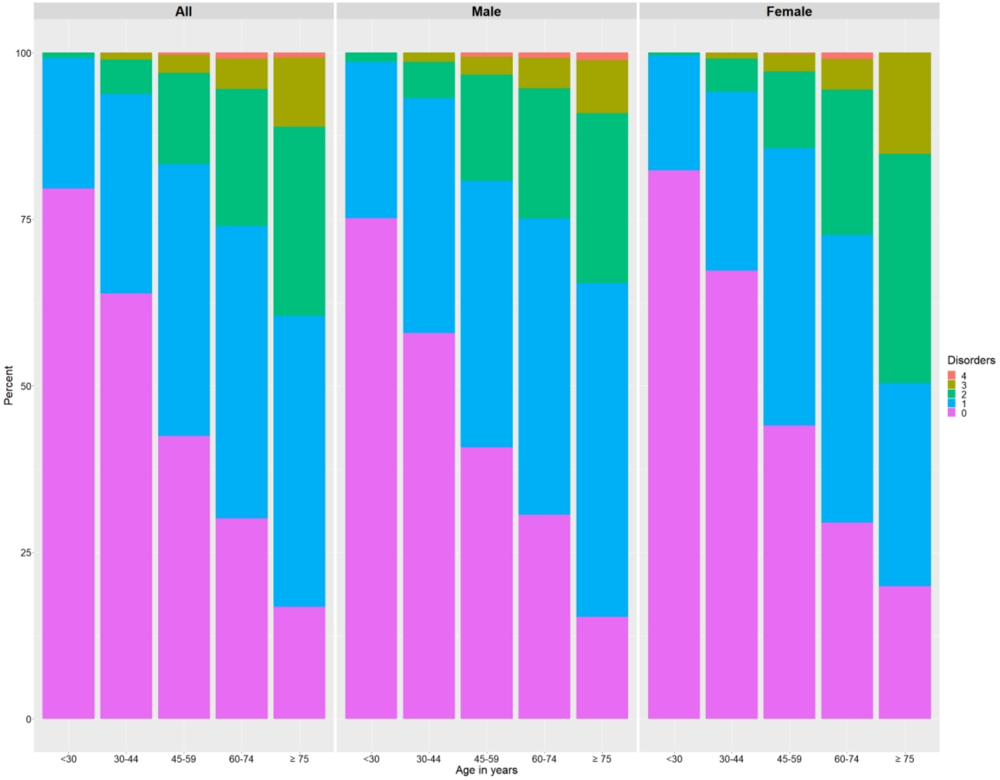

Fig 2. Clustering of NCDs by gender

237x184mm (300 x 300 DPI)

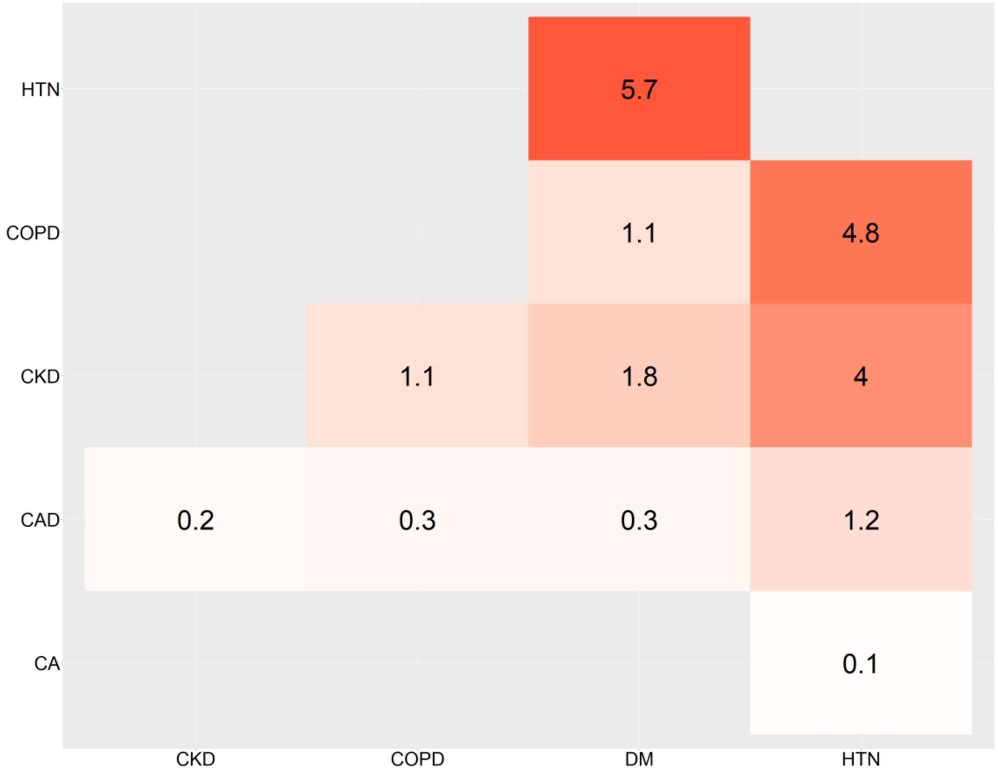

Fig 3. Co-occurrence of NCDs

237x184mm (300 x 300 DPI)

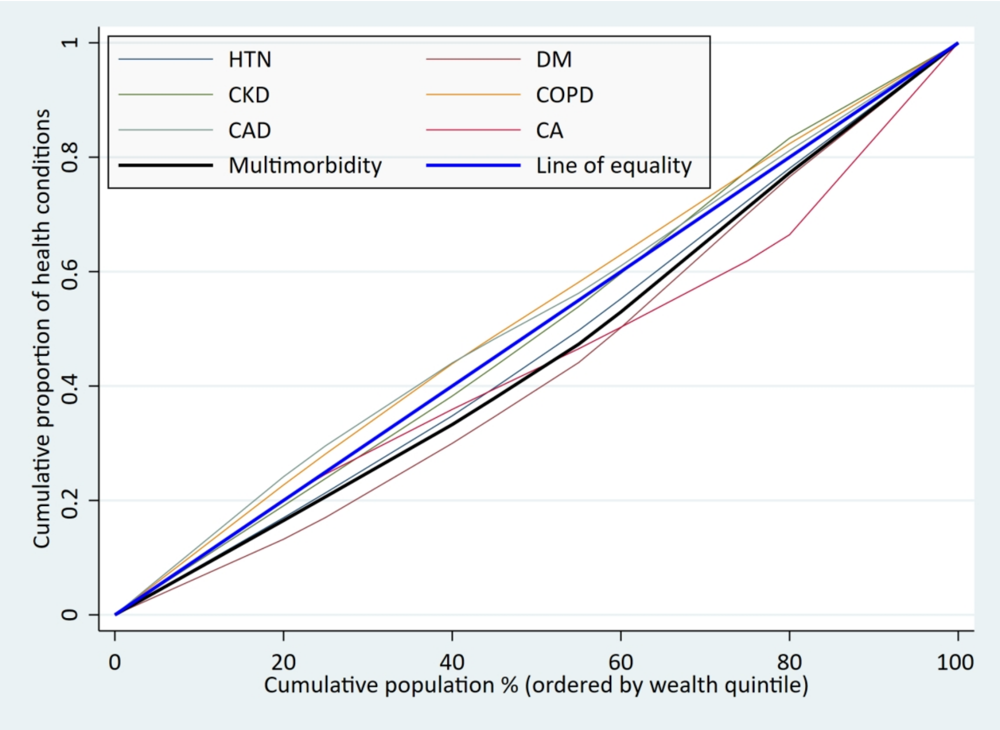

Fig 4. Concentration curves

172x125mm (300 x 300 DPI)

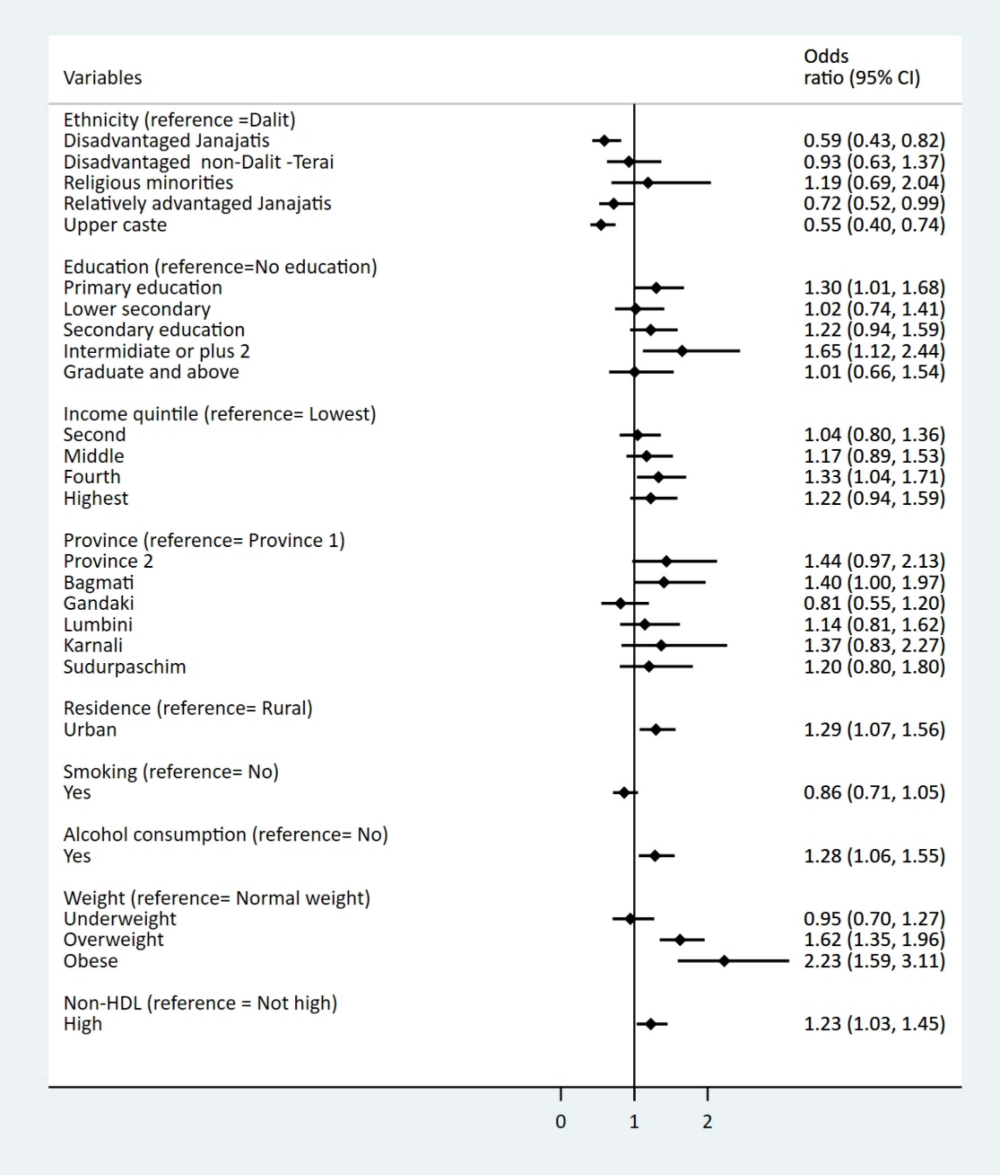

Fig 5. Factors associated with multimorbidity

106x125mm (300 x 300 DPI)

Operational definitions of the key study variables

Variables	Definitions
Smoking	Participants were asked with two questions 'Have you smoked at least 100 cigarettes in your entire life?" and "Do you now smoke cigarettes every day, some days, or not at all?" Smoking at least 100 cigarettes in their lifetime and who, at the time of the survey, smoke either every day or some days was considered smoking (current).
Alcohol	Consuming alcohol in the past 30 days was considered alcohol consumption (current)
Bodyweight	**Body mass index (BMI)** was categorized as underweight (BMI<18.5) normal (BMI 18.0–24.9 kg/m^2), overweight (BMI 25.0–29.9 kg/m^2), or obesity (BMI ≥30 kg/m^2)
Non-HDL	An optimal level of non-HDL cholesterol is considered as having less than 130 milligrams per deciliter (mg/dL)
Hypertension	**Hypertension was** defined as having systolic blood pressure ≥140 mm Hg and/or diastolic blood pressure ≥90 mm Hg during the study, or being previously diagnosed as having hypertension determined by sighting documentation such as a treatment record book or by the history of the participant taking medicine for high blood pressure
Diabetes	Raised fasting glucose (≥126mg/dl) and/or raised postprandial blood glucose level ((≥200mg/dl) and/or if there is the current use of medications for diabetes
Coronary artery diseases (CAD)	Coronary artery disease will be defined as<br>(a) Definite CAD based on any of: documented evidence of prior acute coronary syndrome (ACS) or treatment for CAD, documented history of undergoing coronary angioplasty or CABG, more than 50 % epicardial coronary stenosis by invasive coronary angiography, ECG showing pathological Q waves (any of Minnesota code 1-1-1 to 1-1-7 or 1-2-1 to 1-2-5 or 1-2-7), imaging evidence of a region of loss of viable myocardium that is thinned and has a motion abnormality, in the absence of a non-ischemic cause, RAQ angina plus ECG changes (any of Minnesota codes 4-1-1, 4-1-2, 4-2 or 5-l, 5-2), or RAQ angina plus positive treadmill ECG (exercise-induced horizontal or down-sloping ST depression of ≥ 1 mm at 80 ms from J point), or inducible ischemia on stress imaging;<br>(b) Probable CAD based on any of (in the absence of any of the definite criteria): RAQ angina without significant ECG changes, ECG changes (any of Minnesota Code 4-1-1,4-1-2, 4-2 or 5-1, 5-2) without RAQ angina, or positive treadmill ECG without RAQ angina.<br>(c) Any CAD as those who satisfied either definite or probable CAD criteria.
Chronic Obstructive Pulmonary Disease (COPD)	COPD is the presence of a post-bronchodilator FEV_1/ FVC < 0.70
Chronic Kidney	CKD is defined as glomerular filtration rate <60ml/min/1.73m^2 for three months or more irrespective of the cause.<br>or

Disease (CKD)	by the presence of albuminuria; urinary albumin: creatinine ratio > 30mg/gm to 30 to 300 mg/ gm in two of the three (1 to 2 weeks apart) spot urine specimens
Cancer (CA)	Self-reported cases of any types of cancer

For peer review only

Table 1. Clustering of NCD risk factors by socio-demographic characteristics (multivariable Poisson regression analysis)

Variables		Adjusted prevalence ratio	*p* -value	95% CI
Gender	Female			
	Male	1.13	0.00	1.05 - 1.23
Age groups	<30 years			
	30-44 years	1.92	0.00	1.63 - 2.26
	45-59 years	3.21	0.00	2.73 - 3.79
	60-74 years	4.26	0.00	3.60 - 5.04
	≥75 years	5.50	0.00	4.56 - 6.64
Marital status	Never married			
	Currently married/Cohabiting	0.91	0.45	0.73 - 1.15
	Separated/Divorced	0.75	0.18	0.48 - 1.15
	Widowed	0.92	0.53	0.72 - 1.19
Ethnicity	Dalit			
	Disadvantaged Janajatis	1.29	0.00	1.17 - 1.43
	Disadvantaged non-Dalit Terai caste groups	1.08	0.10	0.99 - 1.18
	Religious minorities	1.28	0.00	1.12 - 1.47
	Relatively advantaged Janajatis	1.20	0.15	0.94 - 1.55
	Upper caste groups	1.15	0.00	1.06 - 1.26
Religion	Hindu			
	Buddhist	0.93	0.30	0.81 - 1.07
	Muslim	1.12	0.27	0.91 - 1.38
	Christian	1.03	0.84	0.77 - 1.39
	Kirat	1.03	0.81	0.82 - 1.29
Education	No eduaction			
	primary education	1.12	0.11	0.97 - 1.29
	lower secondary education	1.16	0.05	1.00 - 1.35
	secondary education	1.15	0.10	0.97 - 1.35
	intermidiate or plus 2	1.08	0.29	0.94 - 1.25
	graduate and above	1.11	0.17	0.96 - 1.30
Occupation	Government employee			
	Non-government employee	0.89	0.29	0.72 - 1.11
	Self-employed/ Business	0.92	0.36	0.77 - 1.10
	Agriculture (commercial)	0.82	0.03	0.69 - 0.98
	Labour	0.78	0.02	0.63 - 0.96
	Student	0.74	0.19	0.46 - 1.17
	Homemaker	0.94	0.49	0.79 - 1.12
	Unemployed	1.00	0.98	0.82 - 1.21
	Retired	1.00	0.98	0.83 - 1.20
Income quintile	Lowest			
	Second	1.01	0.87	0.92 - 1.10
	Middle	1.04	0.40	0.94 - 1.16
	Fourth	1.11	0.03	1.01 - 1.22

For peer review only

	Highest	1.04	0.41	0.95 - 1.14
Province	Province 1			
	Province 2	1.21	0.01	1.04 - 1.40
	Bagmati	1.18	0.01	1.04 - 1.34
	Gandaki	1.04	0.53	0.91 - 1.19
	Lumbini	1.17	0.02	1.02 - 1.33
	Karnali	1.23	0.05	1.00 - 1.50
	Sudurpaschim	1.13	0.16	0.95 - 1.34
Residence	Rural			
	Urban	1.11	0.00	1.04 - 1.20
Smoking	No			
	Yes	0.97	0.41	0.90 - 1.04
ohol consumption	No			
	Yes	1.13	0.00	1.05 - 1.20
Weight	Underweight (<18.5 kg/m2)			
	Normal (18.5-24.9 kg/m2)	0.93	0.17	0.84 - 1.03
	Overweight (25-29.9 kg/m2)	1.27	0.00	1.18 - 1.36
	Obese (≥30 kg/m2)	1.44	0.00	1.28 - 1.61
High non-HDL	No (<130 mg/dL)			
	Yes (≥130 mg/dL)	1.16	0.00	1.10 - 1.24

For peer review only

Table 1. Hierarchical logistic regression models

	Model 1		Model 2		Model 3	
	**Odds ratio**	**95% CI**	**Odds ratio**	**95% CI**	**Odds ratio**	**95% CI**
**Level 1- Distant factors**						
Age groups						
<30 years	Reference					
30-44 years	10.17***	5.49-18.86	9.99***	5.38-18.55	9.45***	4.95-18.06
45-59 years	32.23***	17.09-60.76	31.94***	16.94-60.21	27.10***	13.88-52.89
60-74 years	58.54***	30.69-111.66	58.64***	30.70-112.00	52.78***	26.65-104.53
≥75 years	117.52***	60.33-228.91	117.39***	60.31-228.47	106.19***	52.93-213.02
Ethnicity						
Dalit	Reference					
Disadvantaged Janajatis	0.60**	0.44-0.83	0.58***	0.43-0.80	0.59**	0.43-0.82
Disadvantaged non-Dalit Terai cast	0.88	0.61-1.28	0.88	0.60-1.27	0.93	0.63-1.37
Religious minorities	1.15	0.69-1.91	1.18	0.71-1.96	1.19	0.69-2.04
Relatively advantaged Janajatis	0.79	0.57-1.07	0.76	0.56-1.04	0.72*	0.52-0.99
Upper caste groups	0.54***	0.40-0.73	0.55***	0.41-0.75	0.55***	0.40-0.74
Education						
No education	Reference					
Primary education	1.26	0.99-1.61	1.23	0.96-1.57	1.30*	1.01-1.68
Lower secondary education	1.08	0.79-1.47	1.05	0.77-1.44	1.02	0.74-1.41
Secondary education	1.31*	1.01-1.70	1.27	0.98-1.64	1.22	0.94-1.59
Intermediate or plus 2	1.82**	1.25-2.65	1.75**	1.20-2.54	1.65*	1.12-2.44
Graduate and above	1.08	0.72-1.62	1.04	0.69-1.57	1.01	0.66-1.54
Wealth quintile						
Lowest	Reference					
Second	1.08	0.83-1.40	1.06	0.82-1.38	1.04	0.80-1.36
Middle	1.25	0.96-1.61	1.24	0.96-1.61	1.17	0.89-1.53
Fourth	1.42**	1.13-1.80	1.40**	1.10-1.77	1.33*	1.04-1.71
Highest	1.33*	1.04-1.71	1.31*	1.02-1.68	1.22	0.94-1.59
Province						
Province 1	Reference					
Province 2	1.38	0.94-2.02	1.42	0.97-2.08	1.44	0.97-2.13
Bagmati	1.48*	1.06-2.07	1.51*	1.08-2.11	1.4	1.00-1.97
Gandaki	0.88	0.61-1.29	0.89	0.61-1.30	0.81	0.55-1.20
Lumbini	1.18	0.83-1.66	1.18	0.84-1.67	1.14	0.81-1.62
Karnali	1.22	0.73-2.04	1.27	0.76-2.12	1.37	0.83-2.27
Sudurpaschim	1.13	0.75-1.69	1.17	0.79-1.75	1.2	0.80-1.80
Residence						
Rural	Reference					
Urban	1.39***	1.16-1.66	1.39***	1.16-1.66	1.29**	1.07-1.56
**Level 2- Intermediate factors**						
Smoking						
No	Reference					

For peer review only

Yes			0.80*	0.66-0.96	0.86	0.71-1.05
Alcohol Consumption						
No	Reference					
Yes			1.26*	1.04-1.51	1.28*	1.06-1.55
**Level 3- Immediate factors**						
Body weight						
Underweight	Reference					
Normal					1.06	0.79-1.42
Overweight					1.72***	1.26-2.34
Obese					2.35***	1.54-3.59
High non-HDL						
No	Reference					
Yes					1.23*	1.03-1.45

Note: *, p <0.05; **, p<0.01; ***, p<0.001

For peer review only

STROBE Statement—Checklist of items that should be included in reports of ***cross-sectional studies***

	Item No	Recommendation	Page No
**Title and abstract**	1	(*a*) Indicate the study's design with a commonly used term in the title or the abstract	Line 1-2; Page 1
		(*b*) Provide in the abstract an informative and balanced summary of what was done and what was found	Line 20-42, page 2
**Introduction**			
Background/rationale	2	Explain the scientific background and rationale for the investigation being reported	Line 55-82, page 4
Objectives	3	State specific objectives, including any prespecified hypotheses	Line 81-82; page 4
**Methods**			
Study design	4	Present key elements of study design early in the paper	Line 85-95, page 5
Setting	5	Describe the setting, locations, and relevant dates, including periods of recruitment, exposure, follow-up, and data collection	Line 85-95, page 5
Participants	6	(*a*) Give the eligibility criteria, and the sources and methods of selection of participants	Line 90-95, page 5
Variables	7	Clearly define all outcomes, exposures, predictors, potential confounders, and effect modifiers. Give diagnostic criteria, if applicable	Line 97-118, page 5
Data sources/ measurement	8*	For each variable of interest, give sources of data and details of methods of assessment (measurement). Describe comparability of assessment methods if there is more than one group	Line 97-118, page 5
Bias	9	Describe any efforts to address potential sources of bias	n/a
Study size	10	Explain how the study size was arrived at	Line 90-95, page 5
Quantitative variables	11	Explain how quantitative variables were handled in the analyses. If applicable, describe which groupings were chosen and why	Line 97-118, page 5
Statistical methods	12	(*a*) Describe all statistical methods, including those used to control for confounding	Line 120-142, page 6
		(*b*) Describe any methods used to examine subgroups and interactions	Line 120-142, page 6
		(*c*) Explain how missing data were addressed	Line 140-142, page 6
		(*d*) If applicable, describe analytical methods taking account of sampling strategy	Line 120-142, page 6
		(*e*) Describe any sensitivity analyses	Line 140-142, page 6
**Results**			
Participants	13*	(a) Report numbers of individuals at each stage of study—eg numbers potentially eligible, examined for eligibility, confirmed eligible, included in the study, completing follow-up, and analysed	n/a
		(b) Give reasons for non-participation at each stage	n/a
		(c) Consider use of a flow diagram	n/a

For peer review only - http://bmjopen.bmj.com/site/about/guidelines.xhtml

Descriptive data	14*	(a) Give characteristics of study participants (eg demographic, clinical, social) and information on exposures and potential confounders	Line 152-157, page 7
		(b) Indicate number of participants with missing data for each variable of interest	n/a
Outcome data	15*	Report numbers of outcome events or summary measures	Page 7-8
Main results	16	(*a*) Give unadjusted estimates and, if applicable, confounder-adjusted estimates and their precision (eg, 95% confidence interval). Make clear which confounders were adjusted for and why they were included	Line 181-187, Page 10
		(*b*) Report category boundaries when continuous variables were categorized	n/a
		(*c*) If relevant, consider translating estimates of relative risk into absolute risk for a meaningful time period	n/a
Other analyses	17	Report other analyses done—eg analyses of subgroups and interactions, and sensitivity analyses	Page 7-10
**Discussion**			
Key results	18	Summarise key results with reference to study objectives	Page 10-12
Limitations	19	Discuss limitations of the study, taking into account sources of potential bias or imprecision. Discuss both direction and magnitude of any potential bias	Line 275-280, page 13
Interpretation	20	Give a cautious overall interpretation of results considering objectives, limitations, multiplicity of analyses, results from similar studies, and other relevant evidence	Page 10-12
Generalisability	21	Discuss the generalisability (external validity) of the study results	Page 12-13
**Other information**			
Funding	22	Give the source of funding and the role of the funders for the present study and, if applicable, for the original study on which the present article is based	Line 294, page 14

*Give information separately for exposed and unexposed groups.

Note: An Explanation and Elaboration article discusses each checklist item and gives methodological background and published examples of transparent reporting. The STROBE checklist is best used in conjunction with this article (freely available on the Web sites of PLoS Medicine at http://www.plosmedicine.org/, Annals of Internal Medicine at http://www.annals.org/, and Epidemiology at http://www.epidem.com/). Information on the STROBE Initiative is available at www.strobe-statement.org.

For peer review only

For peer review only - http://bmjopen.bmj.com/site/about/guidelines.xhtml

BMJ Open

Prevalence, pattern, and determinants of chronic disease multimorbidity in Nepal: secondary analysis of a national survey

Journal:	*BMJ Open*
Manuscript ID	bmjopen-2020-047665.R1
Article Type:	Original research
Date Submitted by the Author:	19-May-2021
Complete List of Authors:	Dhungana, Raja Ram; Nepal Family Development Foundation<br>Karki, Khem; Tribhuvan University Institute of Medicine<br>Bista, Bihungum; Nepal Health Research Council<br>Pandey, Achyut; Nepal Health Sector Programme 3 / Monitoring, Evaluation and Operational Research, Abt Associates, Kathmandu, Nepal<br>Dhimal, Meghnath; Nepal Health Research Council<br>Maskey, Mahesh; Nepal Public health Foundation
**Primary Subject Heading**:	Public health
Secondary Subject Heading:	Public health
Keywords:	Hypertension < CARDIOLOGY, General diabetes < DIABETES & ENDOCRINOLOGY, Chronic renal failure < NEPHROLOGY, Chronic airways disease < THORACIC MEDICINE, Coronary heart disease < CARDIOLOGY

SCHOLARONE™
Manuscripts

I, the Submitting Author has the right to grant and does grant on behalf of all authors of the Work (as defined in the below author licence), an exclusive licence and/or a non-exclusive licence for contributions from authors who are: i) UK Crown employees; ii) where BMJ has agreed a CC-BY licence shall apply, and/or iii) in accordance with the terms applicable for US Federal Government officers or employees acting as part of their official duties; on a worldwide, perpetual, irrevocable, royalty-free basis to BMJ Publishing Group Ltd ("BMJ") its licensees and where the relevant Journal is co-owned by BMJ to the co-owners of the Journal, to publish the Work in this journal and any other BMJ products and to exploit all rights, as set out in our licence.

The Submitting Author accepts and understands that any supply made under these terms is made by BMJ to the Submitting Author unless you are acting as an employee on behalf of your employer or a postgraduate student of an affiliated institution which is paying any applicable article publishing charge ("APC") for Open Access articles. Where the Submitting Author wishes to make the Work available on an Open Access basis (and intends to pay the relevant APC), the terms of reuse of such Open Access shall be governed by a Creative Commons licence – details of these licences and which Creative Commons licence will apply to this Work are set out in our licence referred to above.

Other than as permitted in any relevant BMJ Author's Self Archiving Policies, I confirm this Work has not been accepted for publication elsewhere, is not being considered for publication elsewhere and does not duplicate material already published. I confirm all authors consent to publication of this Work and authorise the granting of this licence.

For peer review only

Prevalence, pattern, and determinants of chronic disease multimorbidity in Nepal: secondary analysis of a national survey

Raja Ram Dhungana[1*], Khem Karki[2†], Bihungum Bista[3], Achyut Raj Pandey[4], Meghnath Dhimal[3], Mahesh Kumar Maskey[5]

[1]Nepal Family Development Foundation, Kathmandu, Nepal

[2]Institute of Medicine, Tribhuvan University, Kathmandu, Nepal

[3]Nepal Health Research Council, Kathmandu Nepal

[4]Nepal Health Sector Programme 3 / Monitoring, Evaluation and Operational Research, Abt Associates, Kathmandu, Nepal

[5]Nepal Public Health Foundation, Kathmandu, Nepal

[*], Correspondence: Dr Raja Ram Dhungana. Nepal Family Development Foundation, Nepal

Email: raja.dhungana@gmail.com

[†], Co-first author

For peer review only

For peer review only - http://bmjopen.bmj.com/site/about/guidelines.xhtml

ABSTRACT:

Objectives: To assess the prevalence, pattern, and determinants of noncommunicable diseases (NCDs) multimorbidity in Nepal.

Design: Secondary analysis of the data from the NCD survey 2018, which was conducted between 2016-2018.

Setting: The data belong to the nationally representative survey, that selected the study samples from throughout Nepal using multi-stage cluster sampling.

Participants: 8931 participants aged 20 years and older were included in the study.

Primary Outcomes: NCD multimorbidity (occurrence of two or more chronic conditions including hypertension, diabetes, chronic obstructive pulmonary disease, chronic kidney disease, coronary artery disease, and cancer). Descriptive statistics, concentration indices, and odds ratios were computed to assess pattern and determinants of multimorbidity.

Results: Mean (standard deviation) age was 46.7 years (14.9 years). The majority of the participants were female (57.8%), without formal education (53.4%) and from urban areas (51.5%). Multimorbidity was present in 13.96% (95% CI: 12.9-15.1). Hypertension and diabetes coexisted in 5.7%. Age, alcohol consumption, body mass index, non-HDL level and rural-urban setting were significantly associated with multimorbidity.

Conclusion: Multimorbidity was prevalent in particular groups or geographical areas in Nepal suggesting a need for coordinated and integrated NCD care approach for the management of multiplicative co-comorbid conditions.

Keywords: Multimorbidity, comorbidity, hypertension, diabetes, chronic obstructive pulmonary disease, chronic kidney disease, coronary artery disease, cancer, Nepal

For peer review only

STRENGTH

- This study is the first to assess the prevalence, pattern, and determinants of NCD multimorbidity in Nepal
- Data were collected from the national representative sample of 20 years and older adults in Nepal

For peer review only - http://bmjopen.bmj.com/site/about/guidelines.xhtml

LIMITATIONS

- Data relating to cancer are prone to self-reporting bias
- Estimating the multimorbidity from only six NCDs is likely to underestimate its condition in Nepal

For peer review only

For peer review only - http://bmjopen.bmj.com/site/about/guidelines.xhtml

BACKGROUND

Globally, noncommunicable diseases (NCDs) are giving rise to enormous public health challenges. Recently, the co-existence of two and more NCDs (multimorbidity) has escalated the burden of NCDs globally [1-6]. The prevalence of multimorbidity varies from 12% to 95% across different countries [7-9], clustering particularly in socioeconomically deprived areas [10].

Effects of multimorbidity range from an individual to the health system level. In an individual, effects can stretch out from poor physical and mental health outcomes to high treatment burden for the management of multiple chronic diseases [2 11]. Likewise, multiplications of the comorbid conditions could exert enamours pressure to the existing model of healthcare delivery that lacks coordination and integration of NCD services by increasing healthcare costs and workload [8 11 12].

The major NCDs shared the common behavioural and metabolic risk factors such as physical inactivity, smoking, alcohol consumption and low fruit and vegetables and obesity, which are the leading contributors to burden of multimorbidity [13 14]. Evidence shows that various social circumstances associated with income, education, occupation, gender, and ethnicity also determine the distribution of NCDs and multimorbidity [2 9 15 16]. For example, multimorbidity comes about much earlier in life, up to 15 years earlier, among the people belonging to the poor socio-economic status compared to their wealthier counterparts [4 17]. Additionally, ageing, advancement of healthcare, and increasing life expectancy are closely linked with NCDs and thus are also related to multimorbidity [2 15].

Nepal, a lower-middle income country in South Asia (landlocked between China and India), hosts a multi-cultural, multi-ethnic (over 125 caste/ethnic groups), multi-lingual (129 first languages) and multi-religious (over 10 religions) population of 28 million living mostly in rural areas [18 19]. Recent epidemiological surveys indicated an increasing trend of prevalence of chronic conditions including hypertension and diabetes in Nepal [20]. For example, hypertension increased from 21% to 25% between 2007 [21] and 2019 [20], and prevalence of diabetes rose from 3.6% to 5.8% between 2013 [22] and 2019 [20]. While the prevalence of NCDs is in a rising trend, more individuals are likely to acquire multimorbidity. However, no systematic investigation on NCD multimorbidity is available in Nepal. Detail epidemiological information is essential for facilitating health system preparedness and response in terms of prevention and management of multimorbidity.

For peer review only - http://bmjopen.bmj.com/site/about/guidelines.xhtml

Therefore, this study aimed to assess the prevalence, patterns and determinants of multimorbidity in Nepal.

METHODS

Data source, study participants and sampling

We used the data from the first nationally representative population-based noncommunicable diseases survey in Nepal (shortly, NCD survey) [23]. The cross-sectional survey was conducted between 2016 and 2018. The purpose of the NCD survey was to estimate the prevalence of selected NCDs including hypertension (HTN), diabetes (DM), chronic obstructive pulmonary disease (COPD), Chronic kidney disease (CKD), coronary artery disease (CAD), and cancer (CA) in Nepal.

The NCD survey applied a multistage cluster sampling method to select the participants aged 20 years and above from throughout Nepal. Details of the sampling procedure and survey reports are available elsewhere [23 24]. For our study purpose, we included the data from 8931 participants those having information on six NCDs.

Data collection

The NCD survey collected information on HTN, DM, COPD, CKD, CAD and CA. The survey team recorded three readings of systolic and diastolic blood pressure and used the average of the last two measurements of blood pressure to define hypertension as the condition of having systolic blood pressure of ≥140 mmHg and/or diastolic blood pressure of ≥90 mmHg and/or the use of antihypertensive medication. The survey considered the participants as diabetics if they had raised fasting glucose (≥126mg/dl) or raised postprandial blood glucose level (≥200mg/dl) or the participant was on anti-diabetic medication. COPD was diagnosed based on the ratio of FEV1 to FVC and CKD was confirmed using urinary albumin to creatinine ratio (uACR) and/or measurement of serum creatinine/estimated glomerular filtration rate (e-GFR) [23]. For diagnosing CAD, the survey used the a) information on self-reported admission for myocardial infarction, percutaneous coronary angioplasty or coronary artery bypass surgery; b) electrocardiogram test; and c) responses on the Rose Angina questionnaire [25]. Details are given in the survey report [23]. Cancer diagnosis was confirmed as informed by the participants. Details on operational definitions of the study variables are given in the survey report [23] and Supplementary file 1.

The NCD survey also collected information related to socio-demographic characteristics,

For peer review only - http://bmjopen.bmj.com/site/about/guidelines.xhtml

household income, smoking, alcohol consumption, body mass index (BMI) and lipid. We extracted the data both on the outcome (HTN, DM, COPD, CKD, CAD and CA) and study variables (socio-demographic characteristics, smoking, alcohol consumption, overweight/obesity and high non-HDL) and defined them as per the survey criteria to assess the burden of NCD multimorbidity in Nepal. Multimorbidity was defined as the presence of two and more NCDs [10].

Data analysis

We used the NCD survey information on primary sample units, strata and sampling weight to construct the complex survey weight and performed data analysis using STATA software version 16.1 (Stata Corporation, College Station, TX, USA). All estimates are presented with 95% confidence intervals (CIs). Geographic distribution and clustering of NCDs are presented graphically. To better understand the independent effects of covariates on multimorbidity, we considered the number of NCDs that occurred in each participant as count data and applied multiple Poisson regression and reported the adjusted relative risk (aRR) of having x number NCDs.

For multivariable analysis, we created the hierarchy of the variables as level 1 - distant factors (all socio-demographic variables: age, gender, marital status, ethnicity, religion, education, occupation, household income, Province, and residence), level 2 -intermediate factors (behavioural factors: smoking and alcohol consumption) and level 3- immediate factors (metabolic factors: body mass index and non-HDL) and applied hierarchical modelling using a series of stepwise (backward selection, selection criteria: p-value <0.2) logistic regression. For sensitivity analysis, we imputed the 568 missing data on non-HDL using logistic model and replicated the analysis like in the main analysis. A p-value <0.05 is considered as statistically significant.

Ethics

The NCD survey obtained ethical approval from the Ethical Review Board of Nepal Health Research Council (ethical approval ID: nr110/2016). Informed consent was obtained from the study participants ensuring voluntary participation, privacy and confidentiality.

Patient and public involvement

This is the secondary data analysis of the data from the NCD survey. Patients were not involved in the design or conduct of this study, and nor were members of the general public.

For peer review only - http://bmjopen.bmj.com/site/about/guidelines.xhtml

RESULTS

Sociodemographic characteristics

The mean (standard deviation) age was 46.7 years (14.9 years). The majority of the participants were female (57.8%), married (88.5%), Hindu (88.9%), without formal education (53.4%), and from urban areas (51.5%) (Table 1).

Table 1. Characteristics of the participants (N=8931)

Categories		Male (%)	Female (%)	Total (%)
Age groups	<30 years	5.83	9.51	15.34
	30-44 years	10.38	17.87	28.25
	45-59 years	15.79	16.84	32.63
	60-74 years	11.48	9.12	20.60
	≥75 years	2.12	1.05	3.18
Marital status	Never married	2.75	1.85	4.60
	Currently married/cohabiting	41.55	46.93	88.48
	Separated/Divorced	0.15	0.45	0.60
	Widowed	1.15	5.17	6.32
Ethnicity	Dalit	3.94	5.52	9.46
	Disadvantaged Janajatis	9.45	11.29	20.74
	Disadvantaged non-Dalit Terai caste	8.63	8.55	17.18
	Religious minorities	1.53	1.26	2.79
	Relatively advantaged Janajatis	7.90	9.35	17.25
	Upper caste groups	14.16	18.43	32.59
Religion	Hindu	40.27	48.64	88.91
	Buddhist	2.57	2.84	5.41
	Muslim	1.91	1.76	3.67
	Christian	0.32	0.44	0.76
	Kirat and other	0.54	0.71	1.25
Education	No education	17.10	36.25	53.35
	Primary education	7.34	3.93	11.27
	Lower secondary education	5.22	3.42	8.64
	Secondary education	8.31	5.67	13.98
	Intermediate or plus 2	3.59	2.95	6.54
	Graduate and above	4.05	2.18	6.22
Occupation	Government employee	2.72	1.10	3.82
	Non-government employee	2.95	1.31	4.27
	Self-employed/ Business	8.44	3.55	11.99
	Agriculture (commercial)	20.99	16.83	37.82
	Labour	3.13	0.85	3.99
	Student	1.24	0.98	2.23
	Homemaker	1.27	25.96	27.24
	Unemployed	2.10	3.19	5.29
	Retired	2.74	0.62	3.36
Income quintile	Lowest	9.78	12.47	22.25
	Second	8.59	10.38	18.96

For peer review only

For peer review only - http://bmjopen.bmj.com/site/about/guidelines.xhtml

	Middle	6.87	8.61	15.48
	Fourth	10.99	12.29	23.29
	Highest	9.60	10.42	20.02
Province	Province 1	7.49	9.30	16.78
	Province 2	9.37	9.03	18.40
	Bagmati	10.60	12.98	23.58
	Gandaki	3.92	5.51	9.43
	Lumbini	7.91	8.97	16.88
	Karnali	2.12	3.08	5.20
	Sudurpaschim	4.21	5.53	9.74
Residence	Rural	20.97	27.55	48.52
	Urban	21.19	30.29	51.48

Prevalence of NCD multimorbidity

The prevalence of NCD multimorbidity was 13.96% (95% CI: 12.9-15.1). Participants at older ages, males, religious minorities, those without formal education and obese had a higher prevalence of multimorbidity (Table 2).

Table 2. Prevalence of multimorbidity by subgroups (N=8931)

		Multimorbidity	
**Categories**		**Percent**	**95% CI**
Age groups	<30 years	0.81	0.44-1.5
	30-44 years	6.23	5.2-7.45
	45-59 years	16.75	15.1-18.54
	60-74 years	26.01	23.39-28.82
	≥75 years	39.56	32.94-46.6
Gender	Female	12.02	10.77-13.38
	Male	16.29	14.83-17.86
Marital status	Never married	2.89	1.48-5.56
	Currently married/cohabiting	13.9	12.79-15.09
	Separated/Divorced	14.03	6.74-26.93
	Widowed	22.71	18.98-26.92
Ethnicity	Dalit	14.26	11.77-17.17
	Disadvantaged Janajatis	11.4	9.79-13.24
	Disadvantaged non-Dalit Terai caste	14.69	12.36-17.38
	Religious minorities	21.14	15.98-27.41
	Relatively advantaged Janajatis	18.45	15.52-21.79
	Upper caste groups	12.14	10.64-13.83
Religion	Hindu	13.66	12.54-14.87
	Buddhist	15.29	11.53-20
	Muslim	20.06	15.49-25.58
	Christian	9.34	4.17-19.63
	Kirat and others	14.49	8.25-24.21
Education	No education	15.85	14.45-17.36

For peer review only - http://bmjopen.bmj.com/site/about/guidelines.xhtml

	Primary education	14.32	11.9-17.13
	Lower secondary education	10.8	8.38-13.81
	Secondary education	11.91	9.72-14.52
	Intermediate or plus 2	10.99	8.15-14.66
	Graduate and above	9.28	6.87-12.42
Occupation	Government employee	16.69	12.29-22.26
	Non-government employee	12.79	9.07-17.75
	Self-employed/ Business	15.98	12.95-19.56
	Agriculture(commercial)	11.78	10.37-13.34
	Labour	10.23	7.36-14.03
	Student	0.37	0.09-1.47
	Homemaker	14.81	13.01-16.83
	Unemployed	18.27	14.35-22.98
	Retired	29.71	24.55-35.45
Income quintile	Lowest	11.5	9.91-13.32
	Second	11.73	9.97-13.75
	Middle	13.2	11.16-15.55
	Fourth	16.96	14.83-19.32
	Highest	15.87	13.75-18.24
Residence	Rural	10.89	9.69-12.23
	Urban	17.03	15.32-18.89
Smoking (current)	No	13.9	12.71-15.19
	Yes	14.17	12.37-16.19
Alcohol consumption (current)	No	13.28	12.11-14.56
	Yes	15.91	14.08-17.92
Weight	Underweight (<18.5 kg/m2)	12.26	9.82-15.21
	Normal (18.5-24.9 kg/m2)	10.91	9.87-12.05
	Overweight (25-29.9 kg/m2)	20.74	18.32-23.38
	Obese (≥30 kg/m2)	30.28	25.48-35.55
High non-HDL	No (<130 mg/dL)	11.5	10.4-12.7
	Yes (≥130 mg/dL)	18.73	16.87-20.75

In geographic distribution, Bagmati province had the highest proportion of multimorbidity (Fig 1). The prevalence of multimorbidity also varied by urban and rural settings, where more participants from urban than rural had multimorbidity.

Pattern of multimorbidity

Among the participants having multimorbidity, 80.1% had two NCDs, 17.4% were with three NCDs and 2.5% reported having four and more NCDs. The clustering of NCDs significantly varied with age and gender (Fig 2), where NCDs were more likely to occur at old ages and in males (Supplementary file 2).

For peer review only

For peer review only - http://bmjopen.bmj.com/site/about/guidelines.xhtml

The most common pair of co-occurrences NCDs were hypertension and diabetes, followed by hypertension and COPD, and hypertension and CKD (Fig 3). The triad 'hypertension, diabetes and CKD' was present in 1.4%.

Factors associated with multimorbidity

Age was significantly associated with NCD multimorbidity. The odds of having multimorbidity were 9.4 times for 30-44 years, 27.1 times for 45-59 years and 52.8 for 60-74 years compared to below 30 years of age group (Supplementary file 3). The probability of occurring multimorbidity was significantly higher in overweight/obesity, alcohol users, urban areas and those who had high non-HDL compared to others (Fig 4). The magnitude and direction of the estimates from the imputed dataset were similar to the main analysis.

DISCUSSION

Our study demonstrated the high burden of NCD multimorbidity in Nepal particularly affecting the elderly, obese, alcohol users, religious minorities, higher-income group, and urban residents. This is the very first findings on NCD multimorbidity in Nepal, which may inform the stakeholders on its status, patterns and distribution across Nepal.

The prevalence of NCD multimorbidity in our study was 13.96%. The finding is higher than the rate reported in South Asian counterparts, India and Pakistan (9.4%) [26], and Bangladesh (8.4%) [27], consistent with the prevalence reported in Canada (12.9%) [13], Switzerland (14.5%) [28] and China (14%) [29] and lower than that of Australia (47.1%) [30], Denmark (39.7%), Brazil (29%) [31], England (27.2%) [3], and Vietnam (16.4%) [32]. A systematic review that computed a pooled estimate of multimorbidity from 18 high-income countries and 31 low-and middle-income countries had found the prevalence of multimorbidity to be 33.1% [33]. As the prevalence of chronic conditions tends to differ across different countries owing to differences in dietary practices, lifestyle, alcohol consumption, tobacco use and level of physical activity, among others, it is not uncommon for multimorbidity to vary across countries. Some of these disparities could be because of the differences in the methodology used in individual study. For example, prevalence of multimorbidity also depends on the number of diseases considered in the study, population under study and selection of participants.

For peer review only - http://bmjopen.bmj.com/site/about/guidelines.xhtml

The most common pair of co-occurrences NCDs were hypertension and diabetes (5.7%). The prevalence of comorbid diabetes and hypertension was twice than that we reported (2%) in 2013 in Nepal, suggesting an increasing burden of diabetes and hypertension comorbidity over the years [34]. The prevalence of comorbid diabetes and hypertension was found to be 4.5% in India [35] and 3% in Bangladesh [36]. Similarly, a study by Harrison et al in Australia had found that the co-occurrence of hypertension and diabetes was 4.1% [30]. The higher co-occurrence of diabetes and hypertension or the comorbid condition could be because both conditions are more prevalent in society and also share the common risk factors.

The prevalence of multimorbidity was found to be slightly higher among males in our study. The prevalence of multimorbidity was 16% in males and 12% in females. After adjusting age, the difference in prevalence between males and females was not significant. Like our finding, one of the systematic reviews showed no statistically significant difference in the pooled prevalence of multimorbidity by gender though the nine of the studies those were reviewed reported a higher rate of multimorbidity in females than in males [9]. Likewise, the prevalence of multimorbidity was 14.8% in male and 14.3% in female in Switzerland [28], and 17.2% in male and 15.5% in females in Vietnam, indicating no disparity in multimorbidity in-between [32].

Multiple studies conducted in different setting have revealed age as a determinant of multi-morbidity with the condition being more common with increasing age group [5 28 37]. The prevalence of multimorbidity was 0.81% among participants of age below 30 years and 39.5% in participants of age 75 years or above. In a similar study in Australia, the prevalence of multimorbidity was found to be 1.7% in participants of age 20-29 years, 33.6% among participants of age 70-79 years and 37.7% in participants of age 80 years which also indicates that the multimorbidity increases with increasing age [28]. In another study in the USA, the prevalence of multimorbidity was found to be 67% among those above 65 years and 71% among those above 75 years of age [38]. As most of the NCDs are more common in the older age group, there could have been a higher probability of co-occurrence and thus leading to multimorbidity.

We found that urban area and Bagmati province (capital) had a higher prevalence of multimorbidity. This could be linked to the high burden of NCD risk factors in those areas. Recent surveys suggested that smoking, alcohol consumption, physical inactivity, high salt intake, and obesity were more prevalent in Bagmati province and urban areas than others [20

For peer review only - http://bmjopen.bmj.com/site/about/guidelines.xhtml

39]. Likewise, alcohol consumption, one of the well-established NCD risk factors was also significantly associated with multimorbidity.

In our study, the odds of having multimorbidity was approximately 3 folds higher in obese compared to normal-weight participants. Similar findings were also reported in studies by Agborsangaya et al. [14] and Khan et al. [27] where the odds of having multimorbidity was 2 folds higher in obese. A higher risk of multimorbidity among the obese population could be because it is one of the most common risk factors shared among the NCDs. This study also found that the high non-HDL cholesterol, a strong predictor of cardiovascular diseases [40], was also significantly associated with multimorbidity.

This is the first study to assess the burden of multimorbidity in Nepal. Findings from the nationally representative study could have a high policy implication in terms of tackling the growing burden of multimorbidity in Nepal. Our study found that NCD multimorbidity prevalent in Nepal. Higher prevalence of multimorbidity means more frequent hospital consultations and more hospitalizations [3 5 6]. For example, Schneider et al. [6] reported that the patients with three or more chronic conditions utilize 25 times more hospital beds than peers without any chronic conditions. As higher proportion of healthcare encounters are likely to occur because of multimorbidity, appropriate training of the health facilities at peripheral level and development of an effective referral system could be useful in resource constraint setting like Nepal.

Likewise, with the growing burden of multimorbidity, health system also needs to prepare for dealing with an additional number of health facility visits in Nepal. The current health system organization largely focuses on preventing and managing maternal and child health conditions. It requires a degree of reorganization to deal with the increasing burden of NCDs and multimorbidity. There are several evidence-based models are in practice for the management of multimorbidity globally. Sustainable intEgrated chronic care modeLs for multi-morbidity: delivery, FInancing, and performance (SELFIE) is an example of an innovative framework that suggests the integration of care at micro, meso and macro level according to six pillars of health system: service delivery, leadership and governance, workforce, financing, technologies and medical products and information and research [41]. The strategies, however, should be specific to the Nepalese context and backed by evidence

For peer review only - http://bmjopen.bmj.com/site/about/guidelines.xhtml

on health benefit and cost-effectiveness as the most common comorbid conditions could vary from one country to another.

This study has some limitations. The use of self-reported data for cancer diagnosis might have underestimated the prevalence of cancer in the original study, affecting the current study findings. Likewise, our study could not include other several comorbid conditions such as mental disorders and degenerative diseases and possibly underestimated the burden of multimorbidity in Nepal. For that, a national representative survey encompassing the majority of the common NCDs is recommended.

CONCLUSIONS

This study demonstrated that NCD multimorbidity is prevalent and disproportionately distributed across different socio-economic and demographic strata in Nepal. Growing prevalence of multimorbidity indicates an urgency for the health system to prepare and respond to the inevitable health threats and economic burden in Nepal.

COMPETING INTEREST

None declared.

AUTHORS' CONTRIBUTIONS

RRD and KK conceptualized the study. RRD analysed the data. RRD, BB and ARP interpreted the findings and prepared the first draft of the manuscript. KK, MD and MKM interpreted the findings and drafted the manuscript. All authors read and approved the final manuscript.

FUNDING

The authors received no specific grant from any funding agency for this work.

ACKNOWLEDGMENTS

We would like to thank Nepal Health Research Council for sharing the data. We appreciate all the participants for their great contribution to the study by sharing the information.

For peer review only

DATA SHARING STATEMENT

Data used in the study are reposited in Nepal Health Research Council and are available from the council upon a reasonable request to the Council.

REFERENCE

1. Wild S, Roglic G, Green A, Sicree R, King H. Global prevalence of diabetes: estimates for the year 2000 and projections for 2030. Diabetes care 2004;**27**(5):1047-53
2. Koroukian SM, Schiltz NK, Warner DF, et al. Multimorbidity: constellations of conditions across subgroups of midlife and older individuals, and related Medicare expenditures. Journal of comorbidity 2017;**7**(1):33-43
3. Cassell A, Edwards D, Harshfield A, et al. The epidemiology of multimorbidity in primary care: a retrospective cohort study. British Journal of General Practice 2018;**68**(669):e245-e51
4. Huntley AL, Johnson R, Purdy S, Valderas JM, Salisbury C. Measures of multimorbidity and morbidity burden for use in primary care and community settings: a systematic review and guide. The Annals of Family Medicine 2012;**10**(2):134-41
5. Salisbury C, Johnson L, Purdy S, Valderas JM, Montgomery AA. Epidemiology and impact of multimorbidity in primary care: a retrospective cohort study. British Journal of General Practice 2011;**61**(582):e12-e21
6. Schneider KM, O'Donnell BE, Dean D. Prevalence of multiple chronic conditions in the United States' Medicare population. Health and quality of life outcomes 2009;**7**(1):82
7. Garin N, Koyanagi A, Chatterji S, et al. Global multimorbidity patterns: a cross-sectional, population-based, multi-country study. Journals of Gerontology Series A: Biomedical Sciences and Medical Sciences 2016;**71**(2):205-14
8. Oni T, McGrath N, BeLue R, et al. Chronic diseases and multi-morbidity-a conceptual modification to the WHO ICCC model for countries in health transition. BMC public health 2014;**14**(1):1-7
9. Violan C, Foguet-Boreu Q, Flores-Mateo G, et al. Prevalence, determinants and patterns of multimorbidity in primary care: a systematic review of observational studies. PLoS One 2014;**9**(7):e102149 doi: 10.1371/journal.pone.0102149[published Online First: Epub Date]|.
10. Barnett K, Mercer SW, Norbury M, Watt G, Wyke S, Guthrie B. Epidemiology of multimorbidity and implications for health care, research, and medical education: a cross-sectional study. The Lancet 2012;**380**(9836):37-43 doi: 10.1016/s0140-6736(12)60240-2[published Online First: Epub Date]|.
11. Thavorn K, Maxwell CJ, Gruneir A, et al. Effect of socio-demographic factors on the association between multimorbidity and healthcare costs: a population-based, retrospective cohort study. BMJ open 2017;**7**(10):e017264
12. Wolff JL, Starfield B, Anderson G. Prevalence, expenditures, and complications of multiple chronic conditions in the elderly. Archives of internal medicine 2002;**162**(20):2269-76
13. Roberts K, Rao D, Bennett T, Loukine L, Jayaraman G. Prevalence and patterns of chronic disease multimorbidity and associated determinants in Canada. Health

For peer review only - http://bmjopen.bmj.com/site/about/guidelines.xhtml

promotion and chronic disease prevention in Canada: research, policy and practice 2015;**35**(6):87

14. Agborsangaya CB, Ngwakongnwi E, Lahtinen M, Cooke T, Johnson JA. Multimorbidity prevalence in the general population: the role of obesity in chronic disease clustering. BMC Public Health 2013;**13**(1):1161
15. Wagner K-H, Brath H. A global view on the development of non communicable diseases. Preventive Medicine 2012;**54**:S38-S41 doi: https://doi.org/10.1016/j.ypmed.2011.11.012[published Online First: Epub Date]|.
16. Alwan A. *Global status report on noncommunicable diseases 2010*: World Health Organization, 2011.
17. Mercer SW, Zhou Y, Humphris GM, et al. Multimorbidity and socioeconomic deprivation in primary care consultations. The Annals of Family Medicine 2018;**16**(2):127-31
18. The World Bank Group. Population, total - Nepal. Secondary Population, total - Nepal 2020. https://data.worldbank.org/indicator/SP.POP.TOTL?locations=NP.
19. Central Bureau Of Statistics (CBS) Nepal. National population and housing census 2011. Kathmandu: CBS, 2012.
20. Dhimal M, Bista B, Saroj B, et al. Non communicable diseases risk factors: STEPS Survey Nepal 2019: Nepal Health Research Council (NHRC), 2019.
21. World Health Organisation. STEPwise approach to chronic disease risk factor surveillance: 2007 STEPS Survey report. Secondary STEPwise approach to chronic disease risk factor surveillance: 2007 STEPS Survey report 2008. https://www.who.int/ncds/surveillance/steps/nepal/en/.
22. Aryal KK, Neupane S, Mehata S, et al. Non communicable diseases risk factors: STEPS Survey Nepal 2013. Kathmandu, Nepal: Nepal Health Research Council, 2014.
23. Nepal Health Research Council. Population based prevalence of selected non-Communicable diseases in Nepal. Kathmandu, Nepal, 2019.
24. Dhimal M, Karki KB, Sharma SK, et al. Prevalence of Selected Chronic Non-Communicable Diseases in Nepal. J Nepal Health Res Counc 2019;**17**(3):394-401 doi: 10.33314/jnhrc.v17i3.2327[published Online First: Epub Date]|.
25. Poudel M, Bhandari R, Malla G, et al. Rose Angina Questionnaire: Validation in emergency department to detect myocardial infarction in a tertiary hospital of eastern Nepal. Hong Kong Journal of Emergency Medicine 2020;**27**(5):257-61
26. Singh K, Patel SA, Biswas S, et al. Multimorbidity in South Asian adults: prevalence, risk factors and mortality. Journal of public health (Oxford, England) 2019;**41**(1):80-89 doi: 10.1093/pubmed/fdy017[published Online First: Epub Date]|.
27. Khan N, Rahman M, Mitra D, Afsana K. Prevalence of multimorbidity among Bangladeshi adult population: a nationwide cross-sectional study. BMJ Open 2019;**9**(11):e030886 doi: 10.1136/bmjopen-2019-030886[published Online First: Epub Date]|.
28. Rizza A, Kaplan V, Senn O, Rosemann T, Bhend H, Tandjung R. Age-and gender-related prevalence of multimorbidity in primary care: the Swiss FIRE project. BMC family practice 2012;**13**(1):113
29. Ruel G, Levesque J-F, Stocks N, et al. Understanding the evolution of multimorbidity: evidences from the North West Adelaide Health Longitudinal Study (NWAHS). PLoS One 2014;**9**(5):e96291
30. Harrison C, Henderson J, Miller G, Britt H. The prevalence of complex multimorbidity in Australia. Australian and New Zealand journal of public health 2016;**40**(3):239-44

For peer review only - http://bmjopen.bmj.com/site/about/guidelines.xhtml

31. Araujo MEA, Silva MT, Galvao TF, Nunes BP, Pereira MG. Prevalence and patterns of multimorbidity in Amazon Region of Brazil and associated determinants: a cross-sectional study. BMJ open 2018;**8**(11)
32. Ba NV, Minh HV, Quang LB, et al. Prevalence and correlates of multimorbidity among adults in border areas of the Central Highland Region of Vietnam, 2017. Journal of comorbidity 2019;**9**:2235042X19853382
33. Nguyen H, Manolova G, Daskalopoulou C, Vitoratou S, Prince M, Prina AM. Prevalence of multimorbidity in community settings: A systematic review and meta-analysis of observational studies. J Comorb 2019;**9**:2235042X19870934 doi: 10.1177/2235042X19870934[published Online First: Epub Date]|.
34. Pandey AR, Karki KB, Mehata S, et al. Prevalence and determinants of comorbid diabetes and hypertension in Nepal: evidence from non communicable disease risk factors STEPS Survey Nepal 2013. Journal of Nepal Health Research Council 2015;**13**(29):20-25
35. Tripathy JP, Thakur JS, Jeet G, Jain S. Prevalence and determinants of comorbid diabetes and hypertension: Evidence from non communicable disease risk factor STEPS survey, India. Diabetes & Metabolic Syndrome: Clinical Research & Reviews 2017;**11**:S459-S65
36. Biswas T, Townsend N, Islam MS, et al. Association between socioeconomic status and prevalence of non-communicable diseases risk factors and comorbidities in Bangladesh: findings from a nationwide cross-sectional survey. BMJ open 2019;**9**(3):e025538
37. Pefoyo AJK, Bronskill SE, Gruneir A, et al. The increasing burden and complexity of multimorbidity. BMC public health 2015;**15**(1):415
38. Jindai K, Nielson CM, Vorderstrasse BA, Quiñones AR. Peer Reviewed: Multimorbidity and Functional Limitations Among Adults 65 or Older, NHANES 2005–2012. Preventing chronic disease 2016;**13**
39. Ministry of Health, New ERA, ICF. Nepal Demographic and Health Survey 2016. Kathmandu, Nepal: Ministry of Health, Nepal, 2017.
40. Brunner FJ, Waldeyer C, Ojeda F, et al. Application of non-HDL cholesterol for population-based cardiovascular risk stratification: results from the Multinational Cardiovascular Risk Consortium. The Lancet 2019;**394**(10215):2173-83 doi: https://doi.org/10.1016/S0140-6736(19)32519-X[published Online First: Epub Date]|.
41. Leijten FRM, Struckmann V, van Ginneken E, et al. The SELFIE framework for integrated care for multi-morbidity: development and description. Health policy 2018;**122**(1):12-22

FIGURES

Fig 1. Distribution of multimorbidity by province

Fig 2. Clustering of NCDs by gender

Note: The prevalence of NCDs was likely to increase with aging and was comparatively higher in males than females. Interestingly, compared to the males of the same age group, females of age 45 years and younger were less likely to have NCDs, whereas females of age ≥75 years had higher prevalence of living with at least two NCDs (multimorbidity).

Fig 3. Co-occurrence of NCDs

For peer review only - http://bmjopen.bmj.com/site/about/guidelines.xhtml

Note: Co-occurrence of six major NCDs given in percent; HTN, hypertension; DM, diabetes; COPD, chronic obstructive pulmonary disease; CKD, Chronic kidney disease; CAD, coronary artery disease

Fig 4. Factor associated with multimorbidity

Note: Odds ratio were estimated from the hierarchical logistic regression models. Details of output are given on Supplementary file 2.

SUPPLEMENTARY MATERIALS

Supplementary file 1:

Operational definitions. (DOCX 16.9 KB)

Supplementary file 2:

Clustering of NCDs. (xlsx 13 KB)

Supplementary file 3:

Hierarchical logistic regression models. (xlsx 13 KB)

For peer review only

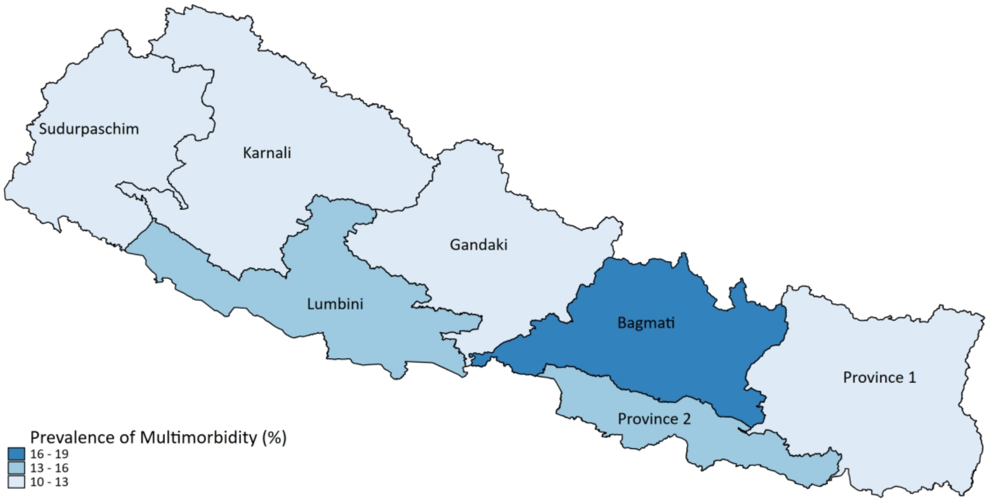

Fig 1. Distribution of multimorbidity by province

242x122mm (300 x 300 DPI)

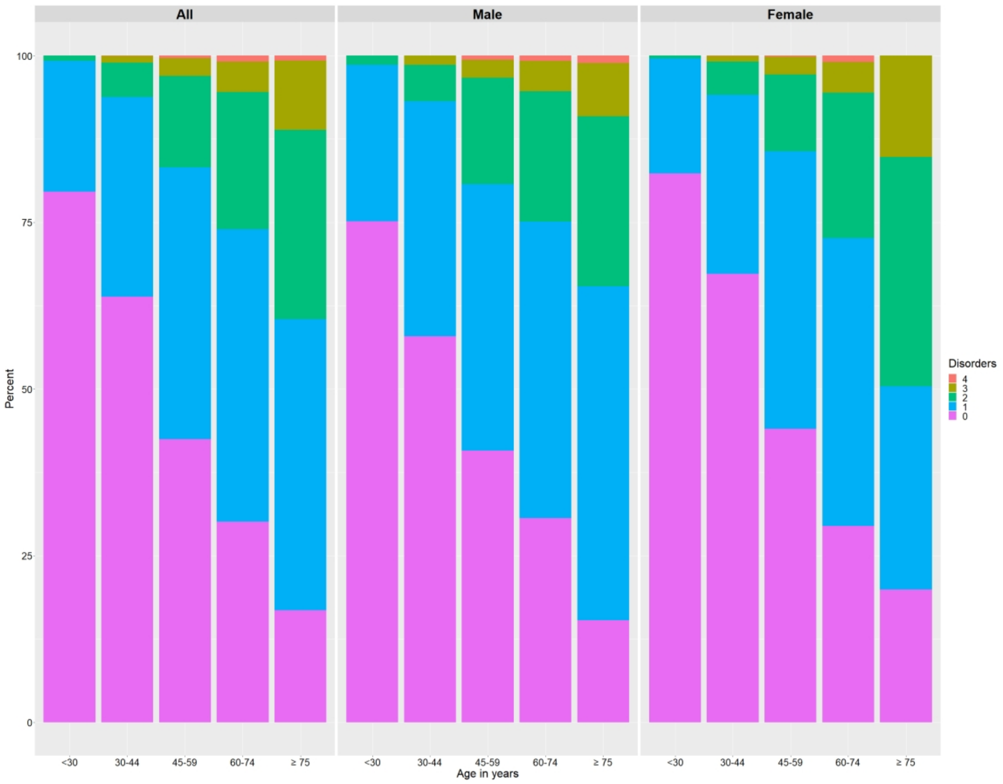

Fig 2. Clustering of NCDs by gender

237x184mm (300 x 300 DPI)

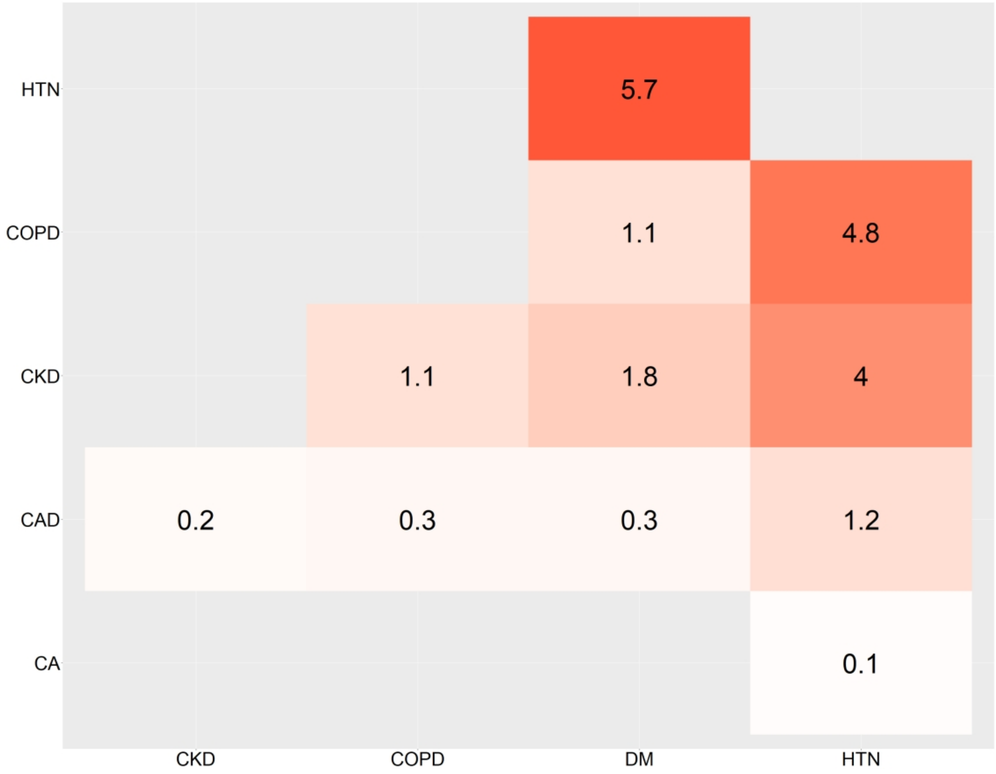

Fig 3. Co-occurrence of NCDs

237x184mm (300 x 300 DPI)

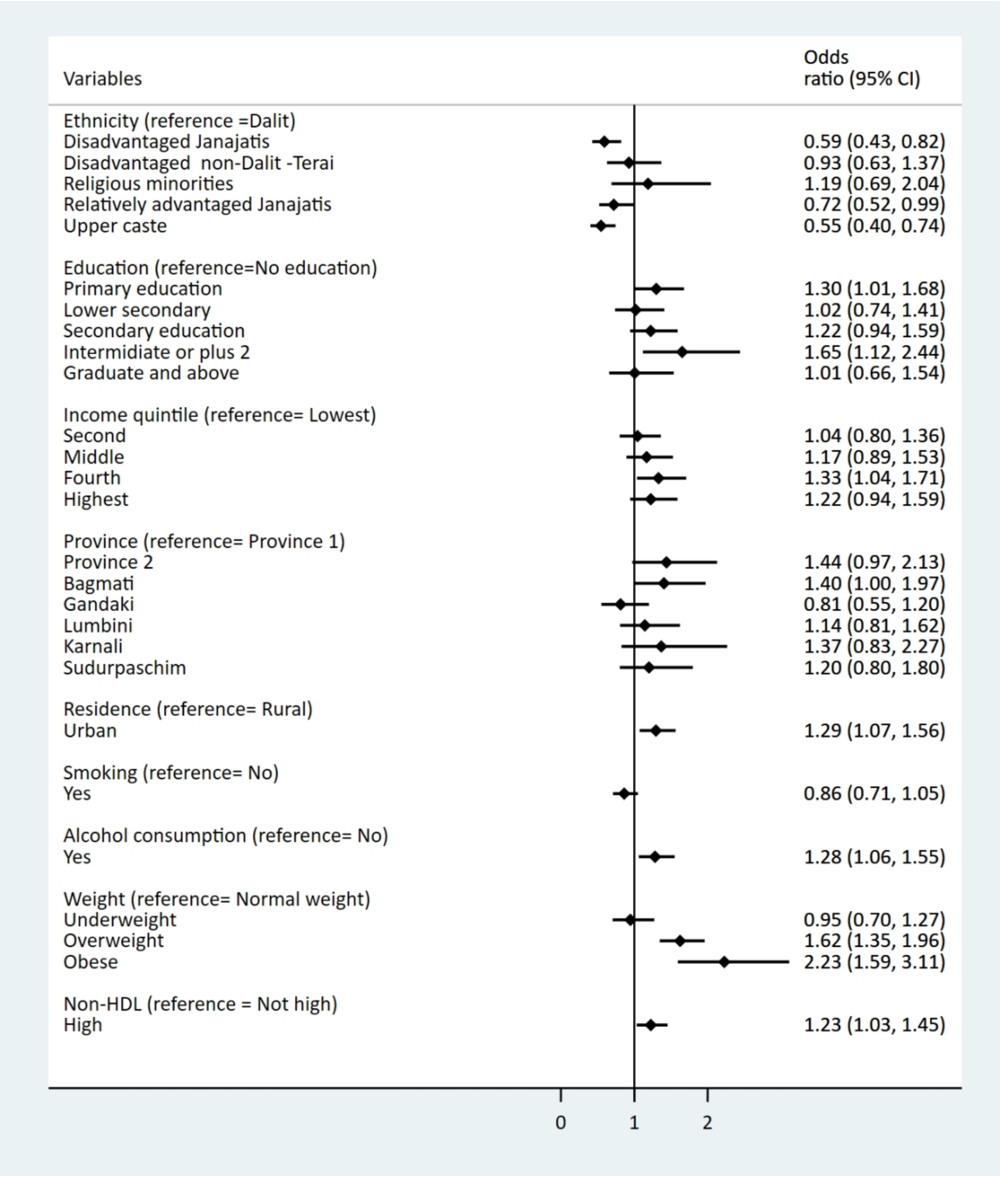

Fig 4. Factor associated with multimorbidity

444x521mm (72 x 72 DPI)

Operational definitions of the key study variables

Variables	Definitions
Smoking	Participants were asked with two questions 'Have you smoked at least 100 cigarettes in your entire life?" and "Do you now smoke cigarettes every day, some days, or not at all?" Smoking at least 100 cigarettes in their lifetime and who, at the time of the survey, smoke either every day or some days was considered smoking (current).
Alcohol	Consuming alcohol in the past 30 days was considered alcohol consumption (current)
Bodyweight	**Body mass index (BMI)** was categorized as underweight (BMI<18.5) normal (BMI 18.0–24.9 kg/m^2), overweight (BMI 25.0–29.9 kg/m^2), or obesity (BMI ≥30 kg/m^2)
Non-HDL	An optimal level of non-HDL cholesterol is considered as having less than 130 milligrams per deciliter (mg/dL)
Hypertension	**Hypertension was** defined as having systolic blood pressure ≥140 mm Hg and/or diastolic blood pressure ≥90 mm Hg during the study, or being previously diagnosed as having hypertension determined by sighting documentation such as a treatment record book or by the history of the participant taking medicine for high blood pressure
Diabetes	Raised fasting glucose (≥126mg/dl) and/or raised postprandial blood glucose level ((≥200mg/dl) and/or if there is the current use of medications for diabetes
Coronary artery diseases (CAD)	Coronary artery disease will be defined as<br>(a) Definite CAD based on any of: documented evidence of prior acute coronary syndrome (ACS) or treatment for CAD, documented history of undergoing coronary angioplasty or CABG, more than 50 % epicardial coronary stenosis by invasive coronary angiography, ECG showing pathological Q waves (any of Minnesota code 1-1-1 to 1-1-7 or 1-2-1 to 1-2-5 or 1-2-7), imaging evidence of a region of loss of viable myocardium that is thinned and has a motion abnormality, in the absence of a non-ischemic cause, RAQ angina plus ECG changes (any of Minnesota codes 4-1-1, 4-1-2, 4-2 or 5-l, 5-2), or RAQ angina plus positive treadmill ECG (exercise-induced horizontal or down-sloping ST depression of ≥ 1 mm at 80 ms from J point), or inducible ischemia on stress imaging;<br>(b) Probable CAD based on any of (in the absence of any of the definite criteria): RAQ angina without significant ECG changes, ECG changes (any of Minnesota Code 4-1-1,4-1-2, 4-2 or 5-1, 5-2) without RAQ angina, or positive treadmill ECG without RAQ angina.<br>(c) Any CAD as those who satisfied either definite or probable CAD criteria.
Chronic Obstructive Pulmonary Disease (COPD)	COPD is the presence of a post-bronchodilator FEV_1/ FVC < 0.70
Chronic Kidney	CKD is defined as glomerular filtration rate <60ml/min/1.73m^2 for three months or more irrespective of the cause.<br>or

Disease (CKD)	by the presence of albuminuria; urinary albumin: creatinine ratio > 30mg/gm to 30 to 300 mg/ gm in two of the three (1 to 2 weeks apart) spot urine specimens
Cancer (CA)	Self-reported cases of any types of cancer

For peer review only

Table 1. Clustering of NCD risk factors by socio-demographic characteristics (multivariable Poisson regression analysis)

Variables		Adjusted prevalence ratio	*p* -value	95% CI
Gender	Female			
	Male	1.13	0.00	1.05 - 1.23
Age groups	<30 years			
	30-44 years	1.92	0.00	1.63 - 2.26
	45-59 years	3.21	0.00	2.73 - 3.79
	60-74 years	4.26	0.00	3.60 - 5.04
	≥75 years	5.50	0.00	4.56 - 6.64
Marital status	Never married			
	Currently married/Cohabiting	0.91	0.45	0.73 - 1.15
	Separated/Divorced	0.75	0.18	0.48 - 1.15
	Widowed	0.92	0.53	0.72 - 1.19
Ethnicity	Dalit			
	Disadvantaged Janajatis	1.29	0.00	1.17 - 1.43
	Disadvantaged non-Dalit Terai caste groups	1.08	0.10	0.99 - 1.18
	Religious minorities	1.28	0.00	1.12 - 1.47
	Relatively advantaged Janajatis	1.20	0.15	0.94 - 1.55
	Upper caste groups	1.15	0.00	1.06 - 1.26
Religion	Hindu			
	Buddhist	0.93	0.30	0.81 - 1.07
	Muslim	1.12	0.27	0.91 - 1.38
	Christian	1.03	0.84	0.77 - 1.39
	Kirat	1.03	0.81	0.82 - 1.29
Education	No eduaction			
	primary education	1.12	0.11	0.97 - 1.29
	lower secondary education	1.16	0.05	1.00 - 1.35
	secondary education	1.15	0.10	0.97 - 1.35
	intermidiate or plus 2	1.08	0.29	0.94 - 1.25
	graduate and above	1.11	0.17	0.96 - 1.30
Occupation	Government employee			
	Non-government employee	0.89	0.29	0.72 - 1.11
	Self-employed/ Business	0.92	0.36	0.77 - 1.10
	Agriculture (commercial)	0.82	0.03	0.69 - 0.98
	Labour	0.78	0.02	0.63 - 0.96
	Student	0.74	0.19	0.46 - 1.17
	Homemaker	0.94	0.49	0.79 - 1.12
	Unemployed	1.00	0.98	0.82 - 1.21
	Retired	1.00	0.98	0.83 - 1.20
Income quintile	Lowest			
	Second	1.01	0.87	0.92 - 1.10
	Middle	1.04	0.40	0.94 - 1.16
	Fourth	1.11	0.03	1.01 - 1.22

For peer review only

	Highest	1.04	0.41	0.95 - 1.14
Province	Province 1			
	Province 2	1.21	0.01	1.04 - 1.40
	Bagmati	1.18	0.01	1.04 - 1.34
	Gandaki	1.04	0.53	0.91 - 1.19
	Lumbini	1.17	0.02	1.02 - 1.33
	Karnali	1.23	0.05	1.00 - 1.50
	Sudurpaschim	1.13	0.16	0.95 - 1.34
Residence	Rural			
	Urban	1.11	0.00	1.04 - 1.20
Smoking	No			
	Yes	0.97	0.41	0.90 - 1.04
ohol consumption	No			
	Yes	1.13	0.00	1.05 - 1.20
Weight	Underweight (<18.5 kg/m2)			
	Normal (18.5-24.9 kg/m2)	0.93	0.17	0.84 - 1.03
	Overweight (25-29.9 kg/m2)	1.27	0.00	1.18 - 1.36
	Obese (≥30 kg/m2)	1.44	0.00	1.28 - 1.61
High non-HDL	No (<130 mg/dL)			
	Yes (≥130 mg/dL)	1.16	0.00	1.10 - 1.24

For peer review only

Table 1. Hierarchical logistic regression models

	Model 1		Model 2		Model 3	
	Odds ratio	95% CI	Odds ratio	95% CI	Odds ratio	95% CI
**Level 1- Distant factors**						
Age groups						
<30 years	Reference					
30-44 years	10.17***	5.49-18.86	9.99***	5.38-18.55	9.45***	4.95-18.06
45-59 years	32.23***	17.09-60.76	31.94***	16.94-60.21	27.10***	13.88-52.89
60-74 years	58.54***	30.69-111.66	58.64***	30.70-112.00	52.78***	26.65-104.53
≥75 years	117.52***	60.33-228.91	117.39***	60.31-228.47	106.19***	52.93-213.02
Ethnicity						
Dalit	Reference					
Disadvantaged Janajatis	0.60**	0.44-0.83	0.58***	0.43-0.80	0.59**	0.43-0.82
Disadvantaged non-Dalit Terai cast	0.88	0.61-1.28	0.88	0.60-1.27	0.93	0.63-1.37
Religious minorities	1.15	0.69-1.91	1.18	0.71-1.96	1.19	0.69-2.04
Relatively advantaged Janajatis	0.79	0.57-1.07	0.76	0.56-1.04	0.72*	0.52-0.99
Upper caste groups	0.54***	0.40-0.73	0.55***	0.41-0.75	0.55***	0.40-0.74
Education						
No education	Reference					
Primary education	1.26	0.99-1.61	1.23	0.96-1.57	1.30*	1.01-1.68
Lower secondary education	1.08	0.79-1.47	1.05	0.77-1.44	1.02	0.74-1.41
Secondary education	1.31*	1.01-1.70	1.27	0.98-1.64	1.22	0.94-1.59
Intermediate or plus 2	1.82**	1.25-2.65	1.75**	1.20-2.54	1.65*	1.12-2.44
Graduate and above	1.08	0.72-1.62	1.04	0.69-1.57	1.01	0.66-1.54
Wealth quintile						
Lowest	Reference					
Second	1.08	0.83-1.40	1.06	0.82-1.38	1.04	0.80-1.36
Middle	1.25	0.96-1.61	1.24	0.96-1.61	1.17	0.89-1.53
Fourth	1.42**	1.13-1.80	1.40**	1.10-1.77	1.33*	1.04-1.71
Highest	1.33*	1.04-1.71	1.31*	1.02-1.68	1.22	0.94-1.59
Province						
Province 1	Reference					
Province 2	1.38	0.94-2.02	1.42	0.97-2.08	1.44	0.97-2.13
Bagmati	1.48*	1.06-2.07	1.51*	1.08-2.11	1.4	1.00-1.97
Gandaki	0.88	0.61-1.29	0.89	0.61-1.30	0.81	0.55-1.20
Lumbini	1.18	0.83-1.66	1.18	0.84-1.67	1.14	0.81-1.62
Karnali	1.22	0.73-2.04	1.27	0.76-2.12	1.37	0.83-2.27
Sudurpaschim	1.13	0.75-1.69	1.17	0.79-1.75	1.2	0.80-1.80
Residence						
Rural	Reference					
Urban	1.39***	1.16-1.66	1.39***	1.16-1.66	1.29**	1.07-1.56
**Level 2- Intermediate factors**						
Smoking						
No	Reference					

For peer review only

Yes			0.80*	0.66-0.96	0.86	0.71-1.05
Alcohol Consumption						
No	Reference					
Yes			1.26*	1.04-1.51	1.28*	1.06-1.55
**Level 3- Immediate factors**						
Body weight						
Underweight	Reference					
Normal					1.06	0.79-1.42
Overweight					1.72***	1.26-2.34
Obese					2.35***	1.54-3.59
High non-HDL						
No	Reference					
Yes					1.23*	1.03-1.45

Note: *, p <0.05; **, p<0.01; ***, p<0.001

For peer review only

STROBE Statement—Checklist of items that should be included in reports of ***cross-sectional studies***

	Item No	Recommendation	Page No
**Title and abstract**	1	(*a*) Indicate the study's design with a commonly used term in the title or the abstract	Line 1-2; Page 1
		(*b*) Provide in the abstract an informative and balanced summary of what was done and what was found	Line 20-42, page 2
**Introduction**			
Background/rationale	2	Explain the scientific background and rationale for the investigation being reported	Line 55-82, page 4
Objectives	3	State specific objectives, including any prespecified hypotheses	Line 81-82; page 4
**Methods**			
Study design	4	Present key elements of study design early in the paper	Line 85-95, page 5
Setting	5	Describe the setting, locations, and relevant dates, including periods of recruitment, exposure, follow-up, and data collection	Line 85-95, page 5
Participants	6	(*a*) Give the eligibility criteria, and the sources and methods of selection of participants	Line 90-95, page 5
Variables	7	Clearly define all outcomes, exposures, predictors, potential confounders, and effect modifiers. Give diagnostic criteria, if applicable	Line 97-118, page 5
Data sources/ measurement	8*	For each variable of interest, give sources of data and details of methods of assessment (measurement). Describe comparability of assessment methods if there is more than one group	Line 97-118, page 5
Bias	9	Describe any efforts to address potential sources of bias	n/a
Study size	10	Explain how the study size was arrived at	Line 90-95, page 5
Quantitative variables	11	Explain how quantitative variables were handled in the analyses. If applicable, describe which groupings were chosen and why	Line 97-118, page 5
Statistical methods	12	(*a*) Describe all statistical methods, including those used to control for confounding	Line 120-142, page 6
		(*b*) Describe any methods used to examine subgroups and interactions	Line 120-142, page 6
		(*c*) Explain how missing data were addressed	Line 140-142, page 6
		(*d*) If applicable, describe analytical methods taking account of sampling strategy	Line 120-142, page 6
		(*e*) Describe any sensitivity analyses	Line 140-142, page 6
**Results**			
Participants	13*	(a) Report numbers of individuals at each stage of study—eg numbers potentially eligible, examined for eligibility, confirmed eligible, included in the study, completing follow-up, and analysed	n/a
		(b) Give reasons for non-participation at each stage	n/a
		(c) Consider use of a flow diagram	n/a

For peer review only - http://bmjopen.bmj.com/site/about/guidelines.xhtml

Descriptive data	14*	(a) Give characteristics of study participants (eg demographic, clinical, social) and information on exposures and potential confounders	Line 152-157, page 7
		(b) Indicate number of participants with missing data for each variable of interest	n/a
Outcome data	15*	Report numbers of outcome events or summary measures	Page 7-8
Main results	16	(*a*) Give unadjusted estimates and, if applicable, confounder-adjusted estimates and their precision (eg, 95% confidence interval). Make clear which confounders were adjusted for and why they were included	Line 181-187, Page 10
		(*b*) Report category boundaries when continuous variables were categorized	n/a
		(*c*) If relevant, consider translating estimates of relative risk into absolute risk for a meaningful time period	n/a
Other analyses	17	Report other analyses done—eg analyses of subgroups and interactions, and sensitivity analyses	Page 7-10
**Discussion**			
Key results	18	Summarise key results with reference to study objectives	Page 10-12
Limitations	19	Discuss limitations of the study, taking into account sources of potential bias or imprecision. Discuss both direction and magnitude of any potential bias	Line 275-280, page 13
Interpretation	20	Give a cautious overall interpretation of results considering objectives, limitations, multiplicity of analyses, results from similar studies, and other relevant evidence	Page 10-12
Generalisability	21	Discuss the generalisability (external validity) of the study results	Page 12-13
**Other information**			
Funding	22	Give the source of funding and the role of the funders for the present study and, if applicable, for the original study on which the present article is based	Line 294, page 14

*Give information separately for exposed and unexposed groups.

Note: An Explanation and Elaboration article discusses each checklist item and gives methodological background and published examples of transparent reporting. The STROBE checklist is best used in conjunction with this article (freely available on the Web sites of PLoS Medicine at http://www.plosmedicine.org/, Annals of Internal Medicine at http://www.annals.org/, and Epidemiology at http://www.epidem.com/). Information on the STROBE Initiative is available at www.strobe-statement.org.

For peer review only

For peer review only - http://bmjopen.bmj.com/site/about/guidelines.xhtml

BMJ Open

Prevalence, pattern, and determinants of chronic disease multimorbidity in Nepal: secondary analysis of a national survey

Journal:	*BMJ Open*
Manuscript ID	bmjopen-2020-047665.R2
Article Type:	Original research
Date Submitted by the Author:	23-Jun-2021
Complete List of Authors:	Dhungana, Raja Ram; Nepal Family Development Foundation<br>Karki, Khem; Tribhuvan University Institute of Medicine<br>Bista, Bihungum; Nepal Health Research Council<br>Pandey, Achyut; Abt Associates<br>Dhimal, Meghnath; Nepal Health Research Council<br>Maskey, Mahesh; Nepal Public Health Foundation
**Primary Subject Heading**:	Public health
Secondary Subject Heading:	Public health
Keywords:	Hypertension < CARDIOLOGY, General diabetes < DIABETES & ENDOCRINOLOGY, Chronic renal failure < NEPHROLOGY, Chronic airways disease < THORACIC MEDICINE, Coronary heart disease < CARDIOLOGY

SCHOLARONE™
Manuscripts

I, the Submitting Author has the right to grant and does grant on behalf of all authors of the Work (as defined in the below author licence), an exclusive licence and/or a non-exclusive licence for contributions from authors who are: i) UK Crown employees; ii) where BMJ has agreed a CC-BY licence shall apply, and/or iii) in accordance with the terms applicable for US Federal Government officers or employees acting as part of their official duties; on a worldwide, perpetual, irrevocable, royalty-free basis to BMJ Publishing Group Ltd ("BMJ") its licensees and where the relevant Journal is co-owned by BMJ to the co-owners of the Journal, to publish the Work in this journal and any other BMJ products and to exploit all rights, as set out in our licence.

The Submitting Author accepts and understands that any supply made under these terms is made by BMJ to the Submitting Author unless you are acting as an employee on behalf of your employer or a postgraduate student of an affiliated institution which is paying any applicable article publishing charge ("APC") for Open Access articles. Where the Submitting Author wishes to make the Work available on an Open Access basis (and intends to pay the relevant APC), the terms of reuse of such Open Access shall be governed by a Creative Commons licence – details of these licences and which Creative Commons licence will apply to this Work are set out in our licence referred to above.

Other than as permitted in any relevant BMJ Author's Self Archiving Policies, I confirm this Work has not been accepted for publication elsewhere, is not being considered for publication elsewhere and does not duplicate material already published. I confirm all authors consent to publication of this Work and authorise the granting of this licence.

For peer review only

Prevalence, pattern, and determinants of chronic disease multimorbidity in Nepal: secondary analysis of a national survey

Raja Ram Dhungana[1*], Khem Karki[2†], Bihungum Bista[3], Achyut Raj Pandey[4], Meghnath Dhimal[3], Mahesh Kumar Maskey[5]

[1]Nepal Family Development Foundation, Kathmandu, Nepal

[2]Institute of Medicine, Tribhuvan University, Kathmandu, Nepal

[3]Nepal Health Research Council, Kathmandu Nepal

[4]Nepal Health Sector Programme 3 / Monitoring, Evaluation and Operational Research, Abt Associates, Kathmandu, Nepal

[5]Nepal Public Health Foundation, Kathmandu, Nepal

[*], Correspondence: Dr Raja Ram Dhungana. Nepal Family Development Foundation, Nepal

Email: raja.dhungana@gmail.com

[†], Co-first author

For peer review only

For peer review only - http://bmjopen.bmj.com/site/about/guidelines.xhtml

ABSTRACT:

Objectives: To assess the prevalence, pattern, and determinants of noncommunicable diseases (NCDs) multimorbidity in Nepal.

Design: Secondary analysis of the data from the NCD survey 2018, which was conducted between 2016-2018.

Setting: The data belong to the nationally representative survey, that selected the study samples from throughout Nepal using multi-stage cluster sampling.

Participants: 8931 participants aged 20 years and older were included in the study.

Primary Outcomes: NCD multimorbidity (occurrence of two or more chronic conditions including hypertension, diabetes, chronic obstructive pulmonary disease, chronic kidney disease, coronary artery disease, and cancer). Descriptive statistics, concentration indices, and odds ratios were computed to assess pattern and determinants of multimorbidity.

Results: Mean (standard deviation) age was 46.7 years (14.9 years). The majority of the participants were female (57.8%), without formal education (53.4%) and from urban areas (51.5%). Multimorbidity was present in 13.96% (95% CI: 12.9-15.1). Hypertension and diabetes coexisted in 5.7%. Age, alcohol consumption, body mass index, non-HDL level and rural-urban setting were significantly associated with multimorbidity.

Conclusion: Multimorbidity was prevalent in particular groups or geographical areas in Nepal suggesting a need for coordinated and integrated NCD care approach for the management of multiplicative co-comorbid conditions.

Keywords: Multimorbidity, comorbidity, hypertension, diabetes, chronic obstructive pulmonary disease, chronic kidney disease, coronary artery disease, cancer, Nepal

STRENGTH

- This study is the first to assess the prevalence, pattern, and determinants of NCD multimorbidity in Nepal
- Data were collected from the national representative sample of 20 years and older adults in Nepal

For peer review only - http://bmjopen.bmj.com/site/about/guidelines.xhtml

47 **LIMITATIONS**

48 - Data relating to cancer are prone to self-reporting bias
49 - Estimating the multimorbidity from only six NCDs is likely to underestimate its
50 condition in Nepal

51

For peer review only

For peer review only - http://bmjopen.bmj.com/site/about/guidelines.xhtml

BACKGROUND

Globally, noncommunicable diseases (NCDs) are giving rise to enormous public health challenges. Recently, the co-existence of two and more NCDs (multimorbidity) has escalated the burden of NCDs globally [1-6]. The prevalence of multimorbidity varies from 12% to 95% across different countries [7-9], clustering particularly in socioeconomically deprived areas [10].

Effects of multimorbidity range from an individual to the health system level. In an individual, effects can stretch out from poor physical and mental health outcomes to high treatment burden for the management of multiple chronic diseases [2 11]. Likewise, multiplications of the comorbid conditions could exert enamours pressure on the existing model of healthcare delivery that lacks coordination and integration of NCD services by increasing healthcare costs and workload [8 11 12].

The major NCDs shared the common behavioural and metabolic risk factors such as physical inactivity, smoking, alcohol consumption and low fruit and vegetables and obesity, which are the leading contributors to burden of multimorbidity [13 14]. Evidence shows that various social circumstances associated with income, education, occupation, gender, and ethnicity also determine the distribution of NCDs and multimorbidity [2 9 15 16]. For example, multimorbidity comes about much earlier in life, up to 15 years earlier, among the people belonging to the poor socio-economic status compared to their wealthier counterparts [4 17]. Additionally, ageing, advancement of healthcare, and increasing life expectancy are closely linked with NCDs and thus are also related to multimorbidity [2 15].

Nepal, a lower-middle income country in South Asia (landlocked between China and India), hosts a multi-cultural, multi-ethnic (over 125 castes/ethnic groups), multi-lingual (129 first languages) and multi-religious (over 10 religions) population of 28 million living mostly in rural areas [18 19]. Recent epidemiological surveys indicated an increasing trend of prevalence of chronic conditions including hypertension and diabetes in Nepal [20]. For example, hypertension increased from 21% to 25% between 2007 [21] and 2019 [20], and prevalence of diabetes rose from 3.6% to 5.8% between 2013 [22] and 2019 [20]. While the prevalence of NCDs is in a rising trend, more individuals are likely to acquire multimorbidity. However, no systematic investigation on NCD multimorbidity is available in Nepal. Detail epidemiological information is essential for facilitating health system preparedness and response in terms of prevention and management of multimorbidity.

For peer review only - http://bmjopen.bmj.com/site/about/guidelines.xhtml

Therefore, this study aimed to assess the prevalence, patterns and determinants of multimorbidity in Nepal.

METHODS

Data source, study participants and sampling

We used the data from the first nationally representative population-based noncommunicable diseases survey in Nepal (shortly, NCD survey) [23]. The cross-sectional survey was conducted between 2016 and 2018. The purpose of the NCD survey was to estimate the prevalence of selected NCDs including hypertension (HTN), diabetes (DM), chronic obstructive pulmonary disease (COPD), Chronic kidney disease (CKD), coronary artery disease (CAD), and cancer (CA) in Nepal.

The NCD survey applied a multistage cluster sampling method to select the participants aged 20 years and above from throughout Nepal. Details of the sampling procedure and survey reports are available elsewhere [23 24]. For our study purpose, we included the data from 8931 participants those having information on six NCDs.

Data collection

The NCD survey collected information on HTN, DM, COPD, CKD, CAD and CA. The survey team recorded three readings of systolic and diastolic blood pressure and used the average of the last two measurements of blood pressure to define hypertension as the condition of having systolic blood pressure of ≥140 mmHg and/or diastolic blood pressure of ≥90 mmHg and/or the use of antihypertensive medication. The survey considered the participants as diabetics if they had raised fasting glucose (≥126mg/dl) or raised postprandial blood glucose level (≥200mg/dl) or the participant was on anti-diabetic medication. COPD was diagnosed based on the ratio of FEV1 to FVC and CKD was confirmed using urinary albumin to creatinine ratio (uACR) and/or measurement of serum creatinine/estimated glomerular filtration rate (e-GFR) [23]. For diagnosing CAD, the survey used the a) information on self-reported admission for myocardial infarction, percutaneous coronary angioplasty or coronary artery bypass surgery; b) electrocardiogram test; and c) responses on the Rose Angina questionnaire [25]. Details are given in the survey report [23]. Cancer diagnosis was confirmed as informed by the participants. Details on operational definitions of the study variables are given in the survey report [23] and Supplementary file 1.

The NCD survey also collected information related to socio-demographic characteristics,

For peer review only - http://bmjopen.bmj.com/site/about/guidelines.xhtml

household income, smoking, alcohol consumption, body mass index (BMI) and lipid. We extracted the data both on the outcome (HTN, DM, COPD, CKD, CAD and CA) and study variables (socio-demographic characteristics, smoking, alcohol consumption, overweight/obesity and high non-HDL) and defined them as per the survey criteria to assess the burden of NCD multimorbidity in Nepal. Multimorbidity was defined as the presence of two and more NCDs [10].

Data analysis

We used the NCD survey information on primary sample units, strata and sampling weight to construct the complex survey weight and performed data analysis using STATA software version 16.1 (Stata Corporation, College Station, TX, USA). All estimates are presented with 95% confidence intervals (CIs). Geographic distribution and clustering of NCDs are presented graphically. To better understand the independent effects of covariates on multimorbidity, we considered the number of NCDs that occurred in each participant as count data and applied multiple Poisson regression and reported the adjusted relative risk (aRR) of having x number NCDs.

For multivariable analysis, we created the hierarchy of the variables as level 1 - distant factors (all socio-demographic variables: age, gender, marital status, ethnicity, religion, education, occupation, household income, Province, and residence), level 2 -intermediate factors (behavioural factors: smoking and alcohol consumption) and level 3- immediate factors (metabolic factors: body mass index and non-HDL) and applied hierarchical modelling using a series of stepwise (backward selection, selection criteria: p-value <0.2) logistic regression. Hierarchical approach in multivariable data analysis is one of the improved methods for estimating the effect of explanatory variables if there are complex hierarchical inter-relationships between these variables [26]. For sensitivity analysis, we imputed the 568 missing data on non-HDL using the logistic model and replicated the analysis like in the main analysis. A p-value <0.05 is considered statistically significant.

Ethics

The NCD survey obtained ethical approval from the Ethical Review Board of Nepal Health Research Council (ethical approval ID: nr110/2016). Informed consent was obtained from the study participants ensuring voluntary participation, privacy and confidentiality.

Patient and public involvement

This is the secondary data analysis of the data from the NCD survey. Patients were not involved in the design or conduct of this study, and nor were members of the general public.

For peer review only - http://bmjopen.bmj.com/site/about/guidelines.xhtml

RESULTS

Sociodemographic characteristics

The mean (standard deviation) age was 46.7 years (14.9 years). The majority of the participants were female (57.8%), married (88.5%), Hindu (88.9%), without formal education (53.4%), and from urban areas (51.5%) (Table 1).

Table 1. Characteristics of the participants (N=8931)

Categories		Male (%)	Female (%)	Total (%)
Age groups	<30 years	5.83	9.51	15.34
	30-44 years	10.38	17.87	28.25
	45-59 years	15.79	16.84	32.63
	60-74 years	11.48	9.12	20.60
	≥75 years	2.12	1.05	3.18
Marital status	Never married	2.75	1.85	4.60
	Currently married/cohabiting	41.55	46.93	88.48
	Separated/Divorced	0.15	0.45	0.60
	Widowed	1.15	5.17	6.32
Ethnicity	Dalit	3.94	5.52	9.46
	Disadvantaged Janajatis	9.45	11.29	20.74
	Disadvantaged non-Dalit Terai caste	8.63	8.55	17.18
	Religious minorities	1.53	1.26	2.79
	Relatively advantaged Janajatis	7.90	9.35	17.25
	Upper caste groups	14.16	18.43	32.59
Religion	Hindu	40.27	48.64	88.91
	Buddhist	2.57	2.84	5.41
	Muslim	1.91	1.76	3.67
	Christian	0.32	0.44	0.76
	Kirat and other	0.54	0.71	1.25
Education	No education	17.10	36.25	53.35
	Primary education	7.34	3.93	11.27
	Lower secondary education	5.22	3.42	8.64
	Secondary education	8.31	5.67	13.98
	Intermediate or plus 2	3.59	2.95	6.54
	Graduate and above	4.05	2.18	6.22
Occupation	Government employee	2.72	1.10	3.82
	Non-government employee	2.95	1.31	4.27
	Self-employed/ Business	8.44	3.55	11.99
	Agriculture (commercial)	20.99	16.83	37.82
	Labour	3.13	0.85	3.99
	Student	1.24	0.98	2.23
	Homemaker	1.27	25.96	27.24
	Unemployed	2.10	3.19	5.29
	Retired	2.74	0.62	3.36

For peer review only - http://bmjopen.bmj.com/site/about/guidelines.xhtml

Income quintile	Lowest	9.78	12.47	22.25
	Second	8.59	10.38	18.96
	Middle	6.87	8.61	15.48
	Fourth	10.99	12.29	23.29
	Highest	9.60	10.42	20.02
Province	Province 1	7.49	9.30	16.78
	Province 2	9.37	9.03	18.40
	Bagmati	10.60	12.98	23.58
	Gandaki	3.92	5.51	9.43
	Lumbini	7.91	8.97	16.88
	Karnali	2.12	3.08	5.20
	Sudurpaschim	4.21	5.53	9.74
Residence	Rural	20.97	27.55	48.52
	Urban	21.19	30.29	51.48

Prevalence of NCD multimorbidity

The prevalence of NCD multimorbidity was 13.96% (95% CI: 12.9-15.1). Participants at older ages, males, religious minorities, those without formal education and obese had a higher prevalence of multimorbidity (Table 2).

Table 2. Prevalence of multimorbidity by subgroups (N=8931)

Categories		Multimorbidity	
		Percent	95% CI
Age groups	<30 years	0.81	0.44-1.5
	30-44 years	6.23	5.2-7.45
	45-59 years	16.75	15.1-18.54
	60-74 years	26.01	23.39-28.82
	≥75 years	39.56	32.94-46.6
Gender	Female	12.02	10.77-13.38
	Male	16.29	14.83-17.86
Marital status	Never married	2.89	1.48-5.56
	Currently married/cohabiting	13.9	12.79-15.09
	Separated/Divorced	14.03	6.74-26.93
	Widowed	22.71	18.98-26.92
Ethnicity	Dalit	14.26	11.77-17.17
	Disadvantaged Janajatis	11.4	9.79-13.24
	Disadvantaged non-Dalit Terai caste	14.69	12.36-17.38
	Religious minorities	21.14	15.98-27.41
	Relatively advantaged Janajatis	18.45	15.52-21.79
	Upper caste groups	12.14	10.64-13.83
Religion	Hindu	13.66	12.54-14.87
	Buddhist	15.29	11.53-20
	Muslim	20.06	15.49-25.58
	Christian	9.34	4.17-19.63

For peer review only

For peer review only - http://bmjopen.bmj.com/site/about/guidelines.xhtml

	Kirat and others	14.49	8.25-24.21
Education	No education	15.85	14.45-17.36
	Primary education	14.32	11.9-17.13
	Lower secondary education	10.8	8.38-13.81
	Secondary education	11.91	9.72-14.52
	Intermediate or plus 2	10.99	8.15-14.66
	Graduate and above	9.28	6.87-12.42
Occupation	Government employee	16.69	12.29-22.26
	Non-government employee	12.79	9.07-17.75
	Self-employed/ Business	15.98	12.95-19.56
	Agriculture (commercial)	11.78	10.37-13.34
	Labour	10.23	7.36-14.03
	Student	0.37	0.09-1.47
	Homemaker	14.81	13.01-16.83
	Unemployed	18.27	14.35-22.98
	Retired	29.71	24.55-35.45
Income quintile	Lowest	11.5	9.91-13.32
	Second	11.73	9.97-13.75
	Middle	13.2	11.16-15.55
	Fourth	16.96	14.83-19.32
	Highest	15.87	13.75-18.24
Residence	Rural	10.89	9.69-12.23
	Urban	17.03	15.32-18.89
Smoking (current)	No	13.9	12.71-15.19
	Yes	14.17	12.37-16.19
Alcohol consumption (current)	No	13.28	12.11-14.56
	Yes	15.91	14.08-17.92
Weight	Underweight (<18.5 kg/m2)	12.26	9.82-15.21
	Normal (18.5-24.9 kg/m2)	10.91	9.87-12.05
	Overweight (25-29.9 kg/m2)	20.74	18.32-23.38
	Obese (≥30 kg/m2)	30.28	25.48-35.55
High non-HDL	No (<130 mg/dL)	11.5	10.4-12.7
	Yes (≥130 mg/dL)	18.73	16.87-20.75

163

164 In geographic distribution, Bagmati province had the highest proportion of
165 multimorbidity (Fig 1). The prevalence of multimorbidity also varied by urban and rural
166 settings, where more participants from urban than rural had multimorbidity.

167 **Pattern of multimorbidity**

168 Among the participants having multimorbidity, 80.1% had two NCDs, 17.4% were with three
169 NCDs and 2.5% reported having four and more NCDs. The clustering of NCDs significantly
170 varied with age and gender (Fig 2), where NCDs were more likely to occur at old ages and in
171 males (Supplementary file 2).

For peer review only - http://bmjopen.bmj.com/site/about/guidelines.xhtml

The most common pair of co-occurrences NCDs were hypertension and diabetes, followed by hypertension and COPD, and hypertension and CKD (Fig 3). The triad ‘hypertension, diabetes and CKD’ was present in 1.4%.

Factors associated with multimorbidity

Age was significantly associated with NCD multimorbidity. The odds of having multimorbidity were 9.4 times for 30-44 years, 27.1 times for 45-59 years and 52.8 for 60-74 years compared to the below 30 years of age group (Supplementary file 3). The probability of occurring multimorbidity was significantly higher in overweight/obesity, alcohol users, urban areas and those who had high non-HDL compared to others (Fig 4). The magnitude and direction of the estimates from the imputed dataset were similar to the main analysis.

DISCUSSION

Our study demonstrated the high burden of NCD multimorbidity in Nepal particularly affecting the elderly, obese, alcohol users, religious minorities, higher-income group, and urban residents. Our study reports the very first findings on NCD multimorbidity in Nepal, which may inform the stakeholders on its status, patterns and distribution across Nepal.

The prevalence of NCD multimorbidity in our study was 13.96%. The finding is higher than the rate reported in South Asian counterparts, India and Pakistan (9.4%) [27], and Bangladesh (8.4%) [28], consistent with the prevalence reported in Canada (12.9%) [13], Switzerland (14.5%) [29] and China (14%) [30] and lower than that of Australia (47.1%) [31], Denmark (39.7%), Brazil (29%) [32], England (27.2%) [3], and Vietnam (16.4%) [33]. A systematic review that computed a pooled estimate of multimorbidity from 18 high-income countries and 31 low-and middle-income countries had found the prevalence of multimorbidity to be 33.1% [34]. As the prevalence of chronic conditions tends to differ across different countries owing to differences in dietary practices, lifestyle, alcohol consumption, tobacco use and level of physical activity, among others, it is not uncommon for multimorbidity to vary across countries. Some of these disparities could be because of the differences in the methodology used in individual study. For example, prevalence of multimorbidity also depends on the number of diseases considered in the study, population under study and selection of participants.

For peer review only - http://bmjopen.bmj.com/site/about/guidelines.xhtml

The most common pair of co-occurrences of NCDs were hypertension and diabetes (5.7%). The prevalence of comorbid diabetes and hypertension was twice as high as that we reported (2%) in 2013 in Nepal, suggesting an increasing burden of diabetes and hypertension comorbidity over the years [35]. The prevalence of comorbid diabetes and hypertension was found to be 4.5% in India [36] and 3% in Bangladesh [37]. Similarly, a study by Harrison et al in Australia had found that the co-occurrence of hypertension and diabetes was 4.1% [31]. The higher co-occurrence of diabetes and hypertension or the comorbid condition could be because both conditions are more prevalent in society and also share common risk factors.

The prevalence of multimorbidity was found to be slightly higher among males in our study. The prevalence of multimorbidity was 16% in males and 12% in females. After adjusting age, the difference in prevalence between males and females was not significant. Like our finding, one of the systematic reviews showed no statistically significant difference in the pooled prevalence of multimorbidity by gender though the nine of the studies that were reviewed reported a higher rate of multimorbidity in females than in males [9]. Likewise, the prevalence of multimorbidity was 14.8% in male and 14.3% in female in Switzerland [29], and 17.2% in male and 15.5% in females in Vietnam, indicating no disparity in multimorbidity in-between [33].

Multiple studies conducted in different setting have revealed age as a determinant of multi-morbidity with the condition being more common with increasing age group [5 29 38]. The prevalence of multimorbidity was 0.81% among participants of age below 30 years and 39.5% among participants of age 75 years or above. In a similar study in Australia, the prevalence of multimorbidity was found to be 1.7% in participants of age 20-29 years, 33.6% among participants of age 70-79 years and 37.7% in participants of age 80 years which also indicates that the multimorbidity increases with increasing age [29]. In another study in the USA, the prevalence of multimorbidity was found to be 67% among those above 65 years and 71% among those above 75 years of age [39]. As most of the NCDs are more common in the older age group, there could have been a higher probability of co-occurrence and thus leading to multimorbidity.

We found that urban area and Bagmati province (capital) had a higher prevalence of multimorbidity. This could be linked to the high burden of NCD risk factors in those areas. Recent surveys suggested that smoking, alcohol consumption, physical inactivity, high salt intake, and obesity were more prevalent in Bagmati province and urban areas than others [20

For peer review only - http://bmjopen.bmj.com/site/about/guidelines.xhtml

40]. Likewise, alcohol consumption, one of the well-established NCD risk factors was also significantly associated with multimorbidity.

In our study, the odds of having multimorbidity was approximately 3 folds higher in obese compared to normal-weight participants. Similar findings were also reported in studies by Agborsangaya et al. [14] and Khan et al. [28] where the odds of having multimorbidity was 2 folds higher in obese. A higher risk of multimorbidity among the obese population could be because it is one of the most common risk factors shared among NCDs. This study also found that high non-HDL cholesterol, a strong predictor of cardiovascular diseases [41], was also significantly associated with multimorbidity.

This is the first study to assess the burden of multimorbidity in Nepal. Findings from the nationally representative study could have a high policy implication in terms of tackling the growing burden of multimorbidity in Nepal. Our study found that NCD multimorbidity prevalent in Nepal. Higher prevalence of multimorbidity means more frequent hospital consultations and more hospitalizations [3 5 6]. For example, Schneider et al. [6] reported that the patients with three or more chronic conditions utilize 25 times more hospital beds than peers without any chronic conditions. As higher proportion of healthcare encounters are likely to occur because of multimorbidity, appropriate training of the health facilities at peripheral level and development of an effective referral system could be useful in resource constraint setting like Nepal.

Likewise, with the growing burden of multimorbidity, health system also needs to prepare for dealing with an additional number of health facility visits in Nepal. The current health system organization largely focuses on preventing and managing maternal and child health conditions. It requires a degree of reorganization to deal with the increasing burden of NCDs and multimorbidity. There are several evidence-based models are in practice for the management of multimorbidity globally. Sustainable intEgrated chronic care modeLs for multi-morbidity: delivery, FInancing, and performance (SELFIE) is an example of an innovative framework that suggests the integration of care at micro, meso and macro level according to six pillars of health system: service delivery, leadership and governance, workforce, financing, technologies and medical products and information and research [42]. The strategies, however, should be specific to the Nepalese context and backed by evidence

For peer review only - http://bmjopen.bmj.com/site/about/guidelines.xhtml

on health benefit and cost-effectiveness as the most common comorbid conditions could vary from one country to another.

This study has some limitations. The use of self-reported data for cancer diagnosis might have underestimated the prevalence of cancer in the original study, affecting the current study findings. Likewise, our study could not include other several comorbid conditions such as mental disorders and degenerative diseases and possibly underestimated the burden of multimorbidity in Nepal. For that, a national representative survey encompassing the majority of the common NCDs is recommended.

CONCLUSIONS

This study demonstrated that NCD multimorbidity is prevalent and disproportionately distributed across different socio-economic and demographic strata in Nepal. Growing prevalence of multimorbidity indicates an urgency for the health system to prepare and respond to the inevitable health threats and economic burden in Nepal.

COMPETING INTEREST

None declared.

AUTHORS' CONTRIBUTIONS

RRD and KK conceptualized the study. RRD analysed the data. RRD, BB and ARP interpreted the findings and prepared the first draft of the manuscript. KK, MD and MKM interpreted the findings and drafted the manuscript. All authors read and approved the final manuscript.

FUNDING

The authors received no specific grant from any funding agency for this work.

ACKNOWLEDGMENTS

We would like to thank Nepal Health Research Council for sharing the data. We appreciate all the participants for their great contribution to the study by sharing the information.

For peer review only

DATA SHARING STATEMENT

Data used in the study are reposited in Nepal Health Research Council and are available from the council upon a reasonable request to the Council.

REFERENCE

1. Wild S, Roglic G, Green A, Sicree R, King H. Global prevalence of diabetes: estimates for the year 2000 and projections for 2030. Diabetes care 2004;**27**(5):1047-53
2. Koroukian SM, Schiltz NK, Warner DF, et al. Multimorbidity: constellations of conditions across subgroups of midlife and older individuals, and related Medicare expenditures. Journal of comorbidity 2017;**7**(1):33-43
3. Cassell A, Edwards D, Harshfield A, et al. The epidemiology of multimorbidity in primary care: a retrospective cohort study. British Journal of General Practice 2018;**68**(669):e245-e51
4. Huntley AL, Johnson R, Purdy S, Valderas JM, Salisbury C. Measures of multimorbidity and morbidity burden for use in primary care and community settings: a systematic review and guide. The Annals of Family Medicine 2012;**10**(2):134-41
5. Salisbury C, Johnson L, Purdy S, Valderas JM, Montgomery AA. Epidemiology and impact of multimorbidity in primary care: a retrospective cohort study. British Journal of General Practice 2011;**61**(582):e12-e21
6. Schneider KM, O'Donnell BE, Dean D. Prevalence of multiple chronic conditions in the United States' Medicare population. Health and quality of life outcomes 2009;**7**(1):82
7. Garin N, Koyanagi A, Chatterji S, et al. Global multimorbidity patterns: a cross-sectional, population-based, multi-country study. Journals of Gerontology Series A: Biomedical Sciences and Medical Sciences 2016;**71**(2):205-14
8. Oni T, McGrath N, BeLue R, et al. Chronic diseases and multi-morbidity-a conceptual modification to the WHO ICCC model for countries in health transition. BMC public health 2014;**14**(1):1-7
9. Violan C, Foguet-Boreu Q, Flores-Mateo G, et al. Prevalence, determinants and patterns of multimorbidity in primary care: a systematic review of observational studies. PloS one 2014;**9**(7):e102149
10. Barnett K, Mercer SW, Norbury M, Watt G, Wyke S, Guthrie B. Epidemiology of multimorbidity and implications for health care, research, and medical education: a cross-sectional study. The Lancet 2012;**380**(9836):37-43
11. Thavorn K, Maxwell CJ, Gruneir A, et al. Effect of socio-demographic factors on the association between multimorbidity and healthcare costs: a population-based, retrospective cohort study. BMJ open 2017;**7**(10):e017264
12. Wolff JL, Starfield B, Anderson G. Prevalence, expenditures, and complications of multiple chronic conditions in the elderly. Archives of internal medicine 2002;**162**(20):2269-76
13. Roberts K, Rao D, Bennett T, Loukine L, Jayaraman G. Prevalence and patterns of chronic disease multimorbidity and associated determinants in Canada. Health promotion and chronic disease prevention in Canada: research, policy and practice 2015;**35**(6):87

For peer review only - http://bmjopen.bmj.com/site/about/guidelines.xhtml

14. Agborsangaya CB, Ngwakongnwi E, Lahtinen M, Cooke T, Johnson JA. Multimorbidity prevalence in the general population: the role of obesity in chronic disease clustering. BMC Public Health 2013;**13**(1):1161

15. Wagner K-H, Brath H. A global view on the development of non communicable diseases. Preventive Medicine 2012;**54**:S38-S41 doi: https://doi.org/10.1016/j.ypmed.2011.11.012[published Online First: Epub Date]|.

16. Alwan A. *Global status report on noncommunicable diseases 2010*: World Health Organization, 2011.

17. Mercer SW, Zhou Y, Humphris GM, et al. Multimorbidity and socioeconomic deprivation in primary care consultations. The Annals of Family Medicine 2018;**16**(2):127-31

18. The World Bank Group. Population, total - Nepal. Secondary Population, total - Nepal 2020. https://data.worldbank.org/indicator/SP.POP.TOTL?locations=NP.

19. Central Bureau Of Statistics (CBS) Nepal. National population and housing census 2011. Kathmandu: CBS, 2012.

20. Dhimal M, Bista B, Saroj B, et al. Non communicable diseases risk factors: STEPS Survey Nepal 2019: Nepal Health Research Council (NHRC), 2019.

21. World Health Organisation. STEPwise approach to chronic disease risk factor surveillance: 2007 STEPS Survey report. Secondary STEPwise approach to chronic disease risk factor surveillance: 2007 STEPS Survey report 2008. https://www.who.int/ncds/surveillance/steps/nepal/en/.

22. Aryal KK, Neupane S, Mehata S, et al. Non communicable diseases risk factors: STEPS Survey Nepal 2013. Kathmandu, Nepal: Nepal Health Research Council, 2014.

23. Nepal Health Research Council. Population based prevalence of selected non-Communicable diseases in Nepal. Kathmandu, Nepal, 2019.

24. Dhimal M, Karki KB, Sharma SK, et al. Prevalence of Selected Chronic Non-Communicable Diseases in Nepal. J Nepal Health Res Counc 2019;**17**(3):394-401 doi: 10.33314/jnhrc.v17i3.2327[published Online First: Epub Date]|.

25. Poudel M, Bhandari R, Malla G, et al. Rose Angina Questionnaire: Validation in emergency department to detect myocardial infarction in a tertiary hospital of eastern Nepal. Hong Kong Journal of Emergency Medicine 2020;**27**(5):257-61

26. Victora CG, Huttly SR, Fuchs SC, Olinto MT. The role of conceptual frameworks in epidemiological analysis: a hierarchical approach. International Journal of Epidemiology 1997;**26**(1):224-27 doi: 10.1093/ije/26.1.224[published Online First: Epub Date]|.

27. Singh K, Patel SA, Biswas S, et al. Multimorbidity in South Asian adults: prevalence, risk factors and mortality. Journal of public health (Oxford, England) 2019;**41**(1):80-89 doi: 10.1093/pubmed/fdy017[published Online First: Epub Date]|.

28. Khan N, Rahman M, Mitra D, Afsana K. Prevalence of multimorbidity among Bangladeshi adult population: a nationwide cross-sectional study. BMJ Open 2019;**9**(11):e030886 doi: 10.1136/bmjopen-2019-030886[published Online First: Epub Date]|.

29. Rizza A, Kaplan V, Senn O, Rosemann T, Bhend H, Tandjung R. Age-and gender-related prevalence of multimorbidity in primary care: the Swiss FIRE project. BMC family practice 2012;**13**(1):113

30. Ruel G, Levesque J-F, Stocks N, et al. Understanding the evolution of multimorbidity: evidences from the North West Adelaide Health Longitudinal Study (NWAHS). PLoS One 2014;**9**(5):e96291

31. Harrison C, Henderson J, Miller G, Britt H. The prevalence of complex multimorbidity in Australia. Australian and New Zealand journal of public health 2016;**40**(3):239-44

For peer review only - http://bmjopen.bmj.com/site/about/guidelines.xhtml

32. Araujo MEA, Silva MT, Galvao TF, Nunes BP, Pereira MG. Prevalence and patterns of multimorbidity in Amazon Region of Brazil and associated determinants: a cross-sectional study. BMJ open 2018;**8**(11)

33. Ba NV, Minh HV, Quang LB, et al. Prevalence and correlates of multimorbidity among adults in border areas of the Central Highland Region of Vietnam, 2017. Journal of comorbidity 2019;**9**:2235042X19853382

34. Nguyen H, Manolova G, Daskalopoulou C, Vitoratou S, Prince M, Prina AM. Prevalence of multimorbidity in community settings: A systematic review and meta-analysis of observational studies. J Comorb 2019;**9**:2235042X19870934 doi: 10.1177/2235042X19870934[published Online First: Epub Date]|.

35. Pandey AR, Karki KB, Mehata S, et al. Prevalence and determinants of comorbid diabetes and hypertension in Nepal: evidence from non communicable disease risk factors STEPS Survey Nepal 2013. Journal of Nepal Health Research Council 2015;**13**(29):20-25

36. Tripathy JP, Thakur JS, Jeet G, Jain S. Prevalence and determinants of comorbid diabetes and hypertension: Evidence from non communicable disease risk factor STEPS survey, India. Diabetes & Metabolic Syndrome: Clinical Research & Reviews 2017;**11**:S459-S65

37. Biswas T, Townsend N, Islam MS, et al. Association between socioeconomic status and prevalence of non-communicable diseases risk factors and comorbidities in Bangladesh: findings from a nationwide cross-sectional survey. BMJ open 2019;**9**(3):e025538

38. Pefoyo AJK, Bronskill SE, Gruneir A, et al. The increasing burden and complexity of multimorbidity. BMC public health 2015;**15**(1):415

39. Jindai K, Nielson CM, Vorderstrasse BA, Quiñones AR. Peer Reviewed: Multimorbidity and Functional Limitations Among Adults 65 or Older, NHANES 2005–2012. Preventing chronic disease 2016;**13**

40. Ministry of Health, New ERA, ICF. Nepal Demographic and Health Survey 2016. Kathmandu, Nepal: Ministry of Health, Nepal, 2017.

41. Brunner FJ, Waldeyer C, Ojeda F, et al. Application of non-HDL cholesterol for population-based cardiovascular risk stratification: results from the Multinational Cardiovascular Risk Consortium. The Lancet 2019;**394**(10215):2173-83 doi: https://doi.org/10.1016/S0140-6736(19)32519-X[published Online First: Epub Date]|.

42. Leijten FRM, Struckmann V, van Ginneken E, et al. The SELFIE framework for integrated care for multi-morbidity: development and description. Health policy 2018;**122**(1):12-22

FIGURES

Fig 1. Distribution of multimorbidity by province

Fig 2. Clustering of NCDs by gender

Note: The prevalence of NCDs was likely to increase with aging and was comparatively higher in males than females. Interestingly, compared to the males of the same age group, females of age 45 years and younger were less likely to have NCDs, whereas females of age ≥75 years had higher prevalence of living with at least two NCDs (multimorbidity).

Fig 3. Co-occurrence of NCDs

For peer review only - http://bmjopen.bmj.com/site/about/guidelines.xhtml

Note: Co-occurrence of six major NCDs given in percent; HTN, hypertension; DM, diabetes; COPD, chronic obstructive pulmonary disease; CKD, chronic kidney disease; CAD, coronary artery disease

Fig 4. Factor associated with multimorbidity

Note: Odds ratio were estimated from the hierarchical logistic regression models. Details of output are given on Supplementary file 2.

SUPPLEMENTARY MATERIALS

Supplementary file 1:

Operational definitions. (DOCX 16.9 KB)

Supplementary file 2:

Clustering of NCDs. (xlsx 13 KB)

Supplementary file 3:

Hierarchical logistic regression models. (xlsx 13 KB)

For peer review only

For peer review only - http://bmjopen.bmj.com/site/about/guidelines.xhtml

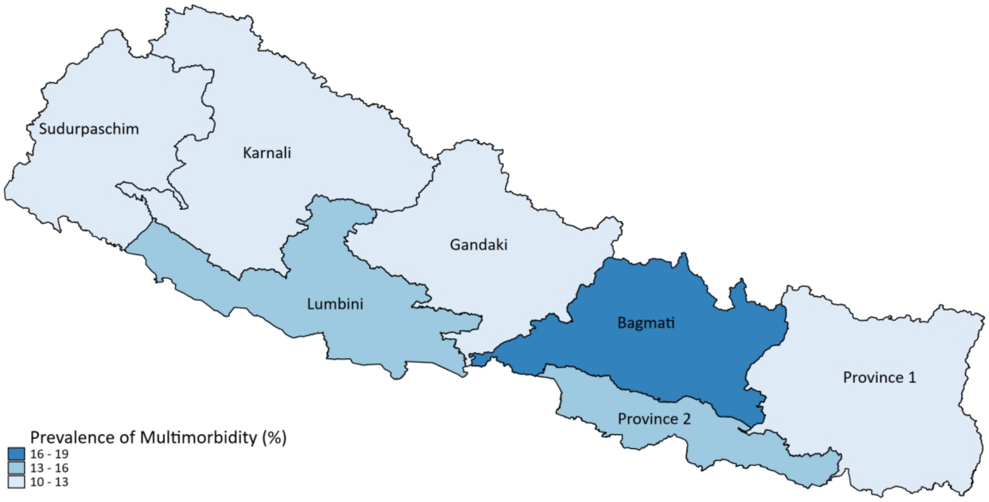

Fig 1. Distribution of multimorbidity by province

242x122mm (300 x 300 DPI)

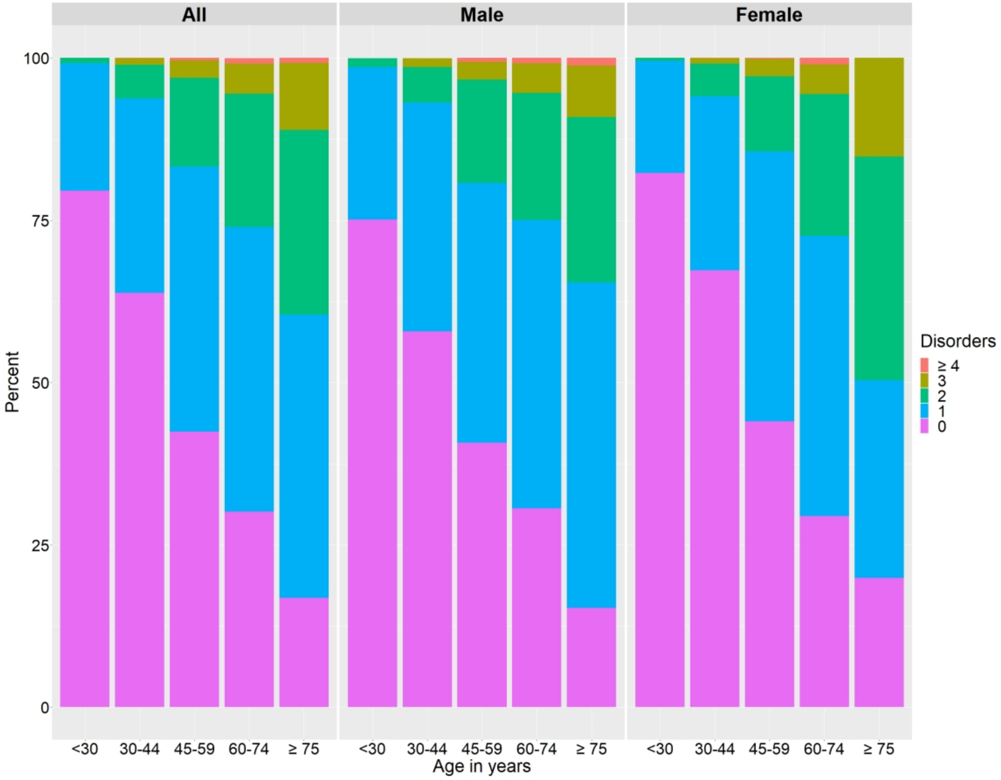

Fig 2. Clustering of NCDs by gender

228x178mm (300 x 300 DPI)

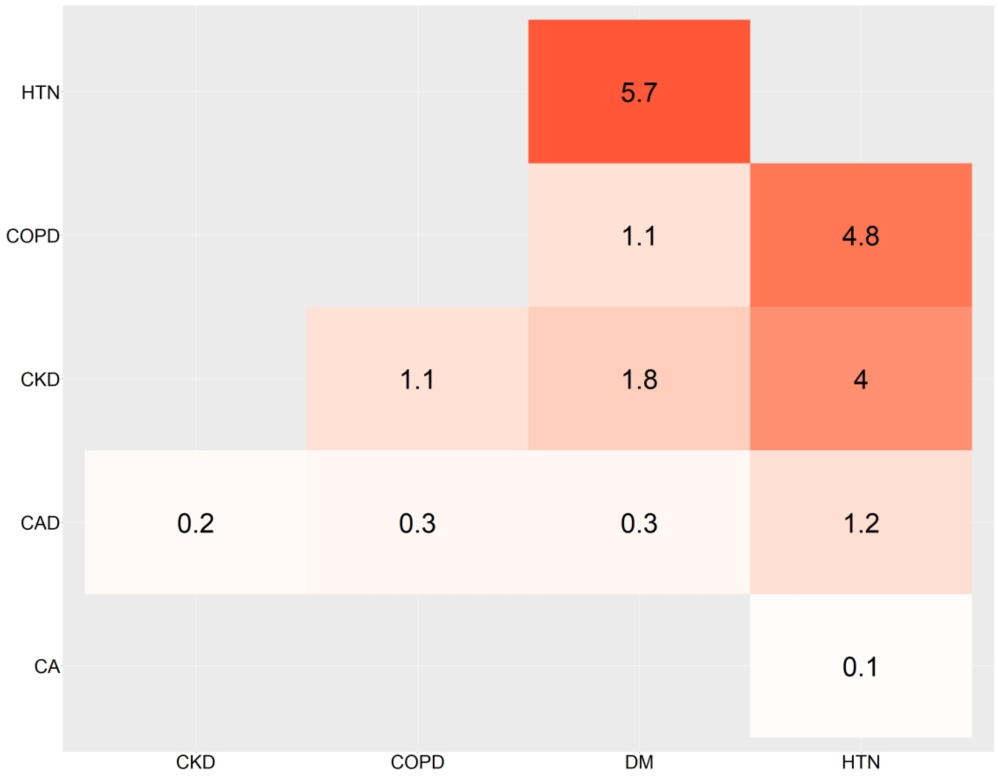

Fig 3. Co-occurrence of NCDs

237x184mm (300 x 300 DPI)

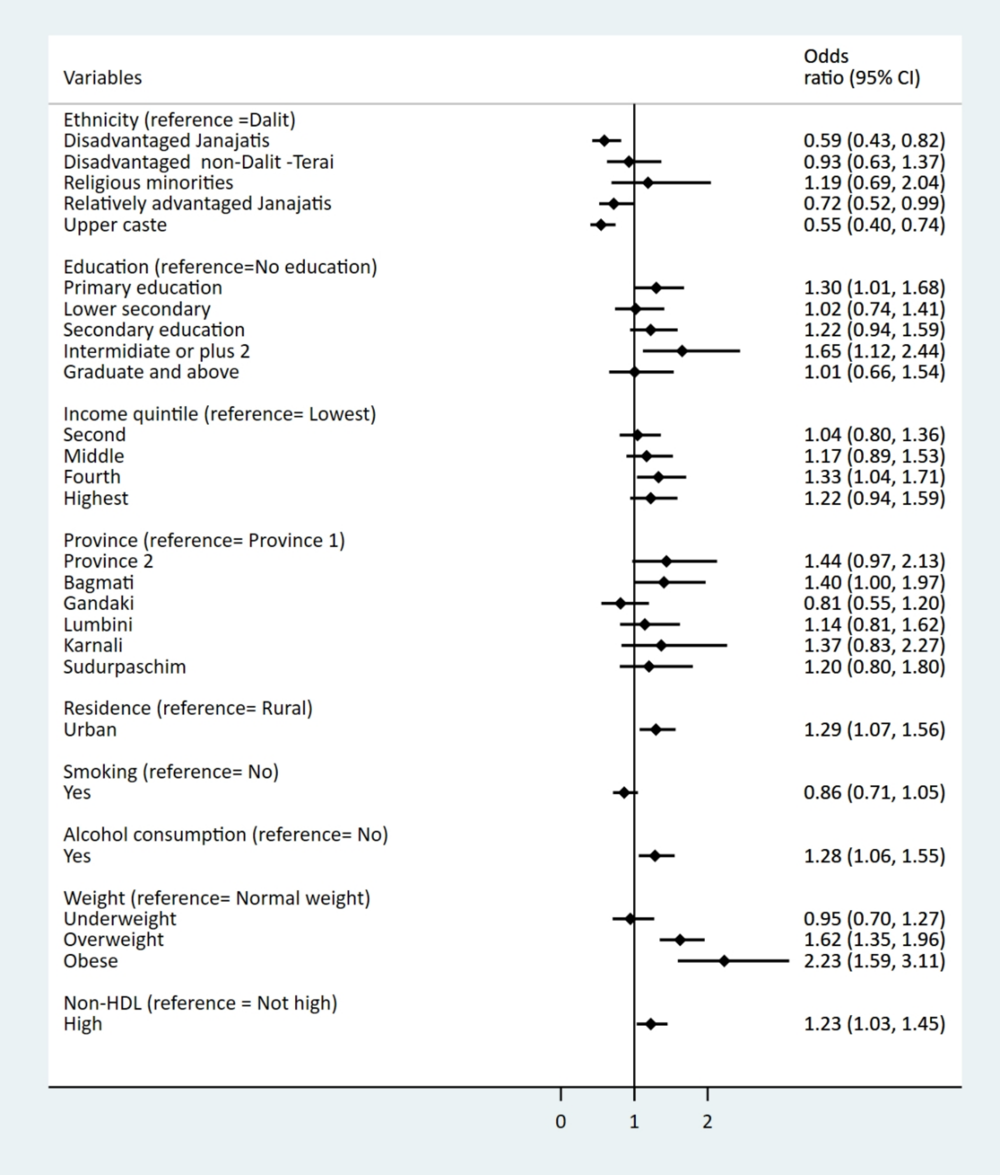

Fig 4. Factors associated with multimorbidity

106x125mm (300 x 300 DPI)

Operational definitions of the key study variables

Variables	Definitions
Smoking	Participants were asked with two questions 'Have you smoked at least 100 cigarettes in your entire life?" and "Do you now smoke cigarettes every day, some days, or not at all?" Smoking at least 100 cigarettes in their lifetime and who, at the time of the survey, smoke either every day or some days was considered smoking (current).
Alcohol	Consuming alcohol in the past 30 days was considered alcohol consumption (current)
Bodyweight	**Body mass index (BMI)** was categorized as underweight (BMI<18.5) normal (BMI 18.0–24.9 kg/m^2), overweight (BMI 25.0–29.9 kg/m^2), or obesity (BMI ≥30 kg/m^2)
Non-HDL	An optimal level of non-HDL cholesterol is considered as having less than 130 milligrams per deciliter (mg/dL)
Hypertension	**Hypertension was** defined as having systolic blood pressure ≥140 mm Hg and/or diastolic blood pressure ≥90 mm Hg during the study, or being previously diagnosed as having hypertension determined by sighting documentation such as a treatment record book or by the history of the participant taking medicine for high blood pressure
Diabetes	Raised fasting glucose (≥126mg/dl) and/or raised postprandial blood glucose level ((≥200mg/dl) and/or if there is the current use of medications for diabetes
Coronary artery diseases (CAD)	Coronary artery disease will be defined as<br>(a) Definite CAD based on any of: documented evidence of prior acute coronary syndrome (ACS) or treatment for CAD, documented history of undergoing coronary angioplasty or CABG, more than 50 % epicardial coronary stenosis by invasive coronary angiography, ECG showing pathological Q waves (any of Minnesota code 1-1-1 to 1-1-7 or 1-2-1 to 1-2-5 or 1-2-7), imaging evidence of a region of loss of viable myocardium that is thinned and has a motion abnormality, in the absence of a non-ischemic cause, RAQ angina plus ECG changes (any of Minnesota codes 4-1-1, 4-1-2, 4-2 or 5-l, 5-2), or RAQ angina plus positive treadmill ECG (exercise-induced horizontal or down-sloping ST depression of ≥ 1 mm at 80 ms from J point), or inducible ischemia on stress imaging;<br>(b) Probable CAD based on any of (in the absence of any of the definite criteria): RAQ angina without significant ECG changes, ECG changes (any of Minnesota Code 4-1-1,4-1-2, 4-2 or 5-1, 5-2) without RAQ angina, or positive treadmill ECG without RAQ angina.<br>(c) Any CAD as those who satisfied either definite or probable CAD criteria.
Chronic Obstructive Pulmonary Disease (COPD)	COPD is the presence of a post-bronchodilator FEV_1/ FVC < 0.70
Chronic Kidney	CKD is defined as glomerular filtration rate <60ml/min/1.73m^2 for three months or more irrespective of the cause.<br>or

Disease (CKD)	by the presence of albuminuria; urinary albumin: creatinine ratio > 30mg/gm to 30 to 300 mg/ gm in two of the three (1 to 2 weeks apart) spot urine specimens
Cancer (CA)	Self-reported cases of any types of cancer

For peer review only

Table 1. Clustering of NCD risk factors by socio-demographic characteristics (multivariable Poisson regression analysis)

Variables		Adjusted prevalence ratio	*p*-value	95% CI
Gender	Female			
	Male	1.13	0.00	1.05 - 1.23
Age groups	<30 years			
	30-44 years	1.92	0.00	1.63 - 2.26
	45-59 years	3.21	0.00	2.73 - 3.79
	60-74 years	4.26	0.00	3.60 - 5.04
	≥75 years	5.50	0.00	4.56 - 6.64
Marital status	Never married			
	Currently married/Cohabiting	0.91	0.45	0.73 - 1.15
	Separated/Divorced	0.75	0.18	0.48 - 1.15
	Widowed	0.92	0.53	0.72 - 1.19
Ethnicity	Dalit			
	Disadvantaged Janajatis	1.29	0.00	1.17 - 1.43
	Disadvantaged non-Dalit Terai caste groups	1.08	0.10	0.99 - 1.18
	Religious minorities	1.28	0.00	1.12 - 1.47
	Relatively advantaged Janajatis	1.20	0.15	0.94 - 1.55
	Upper caste groups	1.15	0.00	1.06 - 1.26
Religion	Hindu			
	Buddhist	0.93	0.30	0.81 - 1.07
	Muslim	1.12	0.27	0.91 - 1.38
	Christian	1.03	0.84	0.77 - 1.39
	Kirat	1.03	0.81	0.82 - 1.29
Education	No education			
	primary education	1.12	0.11	0.97 - 1.29
	lower secondary education	1.16	0.05	1.00 - 1.35
	secondary education	1.15	0.10	0.97 - 1.35
	intermidiate or plus 2	1.08	0.29	0.94 - 1.25
	graduate and above	1.11	0.17	0.96 - 1.30
Occupation	Government employee			
	Non-government employee	0.89	0.29	0.72 - 1.11
	Self-employed/ Business	0.92	0.36	0.77 - 1.10
	Agriculture (commercial)	0.82	0.03	0.69 - 0.98
	Labour	0.78	0.02	0.63 - 0.96
	Student	0.74	0.19	0.46 - 1.17
	Homemaker	0.94	0.49	0.79 - 1.12
	Unemployed	1.00	0.98	0.82 - 1.21
	Retired	1.00	0.98	0.83 - 1.20
Income quintile	Lowest			
	Second	1.01	0.87	0.92 - 1.10
	Middle	1.04	0.40	0.94 - 1.16
	Fourth	1.11	0.03	1.01 - 1.22
	Highest	1.04	0.41	0.95 - 1.14

For peer review only

Province	Province 1			
	Province 2	1.21	0.01	1.04 - 1.40
	Bagmati	1.18	0.01	1.04 - 1.34
	Gandaki	1.04	0.53	0.91 - 1.19
	Lumbini	1.17	0.02	1.02 - 1.33
	Karnali	1.23	0.05	1.00 - 1.50
	Sudurpaschim	1.13	0.16	0.95 - 1.34
Residence	Rural			
	Urban	1.11	0.00	1.04 - 1.20
Smoking	No			
	Yes	0.97	0.41	0.90 - 1.04
ohol consumption	No			
	Yes	1.13	0.00	1.05 - 1.20
Weight	Underweight (<18.5 kg/m2)			
	Normal (18.5-24.9 kg/m2)	0.93	0.17	0.84 - 1.03
	Overweight (25-29.9 kg/m2)	1.27	0.00	1.18 - 1.36
	Obese (≥30 kg/m2)	1.44	0.00	1.28 - 1.61
High non-HDL	No (<130 mg/dL)			
	Yes (≥130 mg/dL)	1.16	0.00	1.10 - 1.24

For peer review only

Table 1. Hierarchical logistic regression models						
	Model 1		Model 2		Model 3	
	Odds ratio	95% CI	Odds ratio	95% CI	Odds ratio	95% CI
**Level 1- Distant factors**						
Age groups						
<30 years	Reference					
30-44 years	10.17***	5.49-18.86	9.99***	5.38-18.55	9.45***	4.95-18.06
45-59 years	32.23***	17.09-60.76	31.94***	16.94-60.21	27.10***	13.88-52.89
60-74 years	58.54***	30.69-111.66	58.64***	30.70-112.00	52.78***	26.65-104.53
≥75 years	117.52***	60.33-228.91	117.39***	60.31-228.47	106.19***	52.93-213.02
Ethnicity						
Dalit	Reference					
Disadvantaged Janajatis	0.60**	0.44-0.83	0.58***	0.43-0.80	0.59**	0.43-0.82
Disadvantaged non-Dalit Terai caste	0.88	0.61-1.28	0.88	0.60-1.27	0.93	0.63-1.37
Religious minorities	1.15	0.69-1.91	1.18	0.71-1.96	1.19	0.69-2.04
Relatively advantaged Janajatis	0.79	0.57-1.07	0.76	0.56-1.04	0.72*	0.52-0.99
Upper caste groups	0.54***	0.40-0.73	0.55***	0.41-0.75	0.55***	0.40-0.74
Education						
No education	Reference					
Primary education	1.26	0.99-1.61	1.23	0.96-1.57	1.30*	1.01-1.68
Lower secondary education	1.08	0.79-1.47	1.05	0.77-1.44	1.02	0.74-1.41
Secondary education	1.31*	1.01-1.70	1.27	0.98-1.64	1.22	0.94-1.59
Intermediate or plus 2	1.82**	1.25-2.65	1.75**	1.20-2.54	1.65*	1.12-2.44
Graduate and above	1.08	0.72-1.62	1.04	0.69-1.57	1.01	0.66-1.54
Wealth quintile						
Lowest	Reference					
Second	1.08	0.83-1.40	1.06	0.82-1.38	1.04	0.80-1.36
Middle	1.25	0.96-1.61	1.24	0.96-1.61	1.17	0.89-1.53
Fourth	1.42**	1.13-1.80	1.40**	1.10-1.77	1.33*	1.04-1.71
Highest	1.33*	1.04-1.71	1.31*	1.02-1.68	1.22	0.94-1.59
Province						
Province 1	Reference					
Province 2	1.38	0.94-2.02	1.42	0.97-2.08	1.44	0.97-2.13
Bagmati	1.48*	1.06-2.07	1.51*	1.08-2.11	1.4	1.00-1.97
Gandaki	0.88	0.61-1.29	0.89	0.61-1.30	0.81	0.55-1.20
Lumbini	1.18	0.83-1.66	1.18	0.84-1.67	1.14	0.81-1.62
Karnali	1.22	0.73-2.04	1.27	0.76-2.12	1.37	0.83-2.27
Sudurpaschim	1.13	0.75-1.69	1.17	0.79-1.75	1.2	0.80-1.80
Residence						
Rural	Reference					
Urban	1.39***	1.16-1.66	1.39***	1.16-1.66	1.29**	1.07-1.56
**Level 2- Intermediate factors**						
Smoking						
No	Reference					

For peer review only

Yes			0.80*	0.66-0.96	0.86	0.71-1.05
Alcohol Consumption						
No	Reference					
Yes			1.26*	1.04-1.51	1.28*	1.06-1.55
**Level 3- Immediate factors**						
Bodyweight						
Underweight	Reference					
Normal					1.06	0.79-1.42
Overweight					1.72***	1.26-2.34
Obese					2.35***	1.54-3.59
High non-HDL						
No	Reference					
Yes					1.23*	1.03-1.45

Note: *, p <0.05; **, p<0.01; ***, p<0.001

For peer review only

STROBE Statement—Checklist of items that should be included in reports of ***cross-sectional studies***

	Item No	Recommendation	Page No
**Title and abstract**	1	(*a*) Indicate the study's design with a commonly used term in the title or the abstract	Line 1-2; Page 1
		(*b*) Provide in the abstract an informative and balanced summary of what was done and what was found	Line 20-42, page 2
**Introduction**			
Background/rationale	2	Explain the scientific background and rationale for the investigation being reported	Line 55-82, page 4
Objectives	3	State specific objectives, including any prespecified hypotheses	Line 81-82; page 4
**Methods**			
Study design	4	Present key elements of study design early in the paper	Line 85-95, page 5
Setting	5	Describe the setting, locations, and relevant dates, including periods of recruitment, exposure, follow-up, and data collection	Line 85-95, page 5
Participants	6	(*a*) Give the eligibility criteria, and the sources and methods of selection of participants	Line 90-95, page 5
Variables	7	Clearly define all outcomes, exposures, predictors, potential confounders, and effect modifiers. Give diagnostic criteria, if applicable	Line 97-118, page 5
Data sources/ measurement	8*	For each variable of interest, give sources of data and details of methods of assessment (measurement). Describe comparability of assessment methods if there is more than one group	Line 97-118, page 5
Bias	9	Describe any efforts to address potential sources of bias	n/a
Study size	10	Explain how the study size was arrived at	Line 90-95, page 5
Quantitative variables	11	Explain how quantitative variables were handled in the analyses. If applicable, describe which groupings were chosen and why	Line 97-118, page 5
Statistical methods	12	(*a*) Describe all statistical methods, including those used to control for confounding	Line 120-142, page 6
		(*b*) Describe any methods used to examine subgroups and interactions	Line 120-142, page 6
		(*c*) Explain how missing data were addressed	Line 140-142, page 6
		(*d*) If applicable, describe analytical methods taking account of sampling strategy	Line 120-142, page 6
		(*e*) Describe any sensitivity analyses	Line 140-142, page 6
**Results**			
Participants	13*	(a) Report numbers of individuals at each stage of study—eg numbers potentially eligible, examined for eligibility, confirmed eligible, included in the study, completing follow-up, and analysed	n/a
		(b) Give reasons for non-participation at each stage	n/a
		(c) Consider use of a flow diagram	n/a

For peer review only - http://bmjopen.bmj.com/site/about/guidelines.xhtml

Descriptive data	14*	(a) Give characteristics of study participants (eg demographic, clinical, social) and information on exposures and potential confounders	Line 152-157, page 7
		(b) Indicate number of participants with missing data for each variable of interest	n/a
Outcome data	15*	Report numbers of outcome events or summary measures	Page 7-8
Main results	16	(*a*) Give unadjusted estimates and, if applicable, confounder-adjusted estimates and their precision (eg, 95% confidence interval). Make clear which confounders were adjusted for and why they were included	Line 181-187, Page 10
		(*b*) Report category boundaries when continuous variables were categorized	n/a
		(*c*) If relevant, consider translating estimates of relative risk into absolute risk for a meaningful time period	n/a
Other analyses	17	Report other analyses done—eg analyses of subgroups and interactions, and sensitivity analyses	Page 7-10
**Discussion**			
Key results	18	Summarise key results with reference to study objectives	Page 10-12
Limitations	19	Discuss limitations of the study, taking into account sources of potential bias or imprecision. Discuss both direction and magnitude of any potential bias	Line 275-280, page 13
Interpretation	20	Give a cautious overall interpretation of results considering objectives, limitations, multiplicity of analyses, results from similar studies, and other relevant evidence	Page 10-12
Generalisability	21	Discuss the generalisability (external validity) of the study results	Page 12-13
**Other information**			
Funding	22	Give the source of funding and the role of the funders for the present study and, if applicable, for the original study on which the present article is based	Line 294, page 14

*Give information separately for exposed and unexposed groups.

Note: An Explanation and Elaboration article discusses each checklist item and gives methodological background and published examples of transparent reporting. The STROBE checklist is best used in conjunction with this article (freely available on the Web sites of PLoS Medicine at http://www.plosmedicine.org/, Annals of Internal Medicine at http://www.annals.org/, and Epidemiology at http://www.epidem.com/). Information on the STROBE Initiative is available at www.strobe-statement.org.

For peer review only
